# A User-Centered Design Approach for a Screening App for People With Cognitive Impairment (digiDEM-SCREEN): Development and Usability Study

**DOI:** 10.2196/65022

**Published:** 2025-01-22

**Authors:** Michael Zeiler, Nikolas Dietzel, Fabian Haug, Julian Haug, Klaus Kammerer, Rüdiger Pryss, Peter Heuschmann, Elmar Graessel, Peter L Kolominsky-Rabas, Hans-Ulrich Prokosch

**Affiliations:** 1 Medical Informatics, Friedrich-Alexander-Universität Erlangen-Nürnberg, Erlangen, Germany; 2 Interdisciplinary Center for Health Technology Assessment and Public Health, Friedrich-Alexander-Universität Erlangen-Nürnberg, Erlangen, Germany; 3 Institute of Clinical Epidemiology and Biometry, Julius-Maximilians-Universität Würzburg, Würzburg, Germany; 4 Institute for Medical Data Science, University Hospital Würzburg, Würzburg, Germany; 5 Clinical Trial Centre Würzburg, University Hospital Würzburg, Würzburg, Germany; 6 Uniklinik Erlangen Department of Psychiatry and Psychotherapy, Center for Health Services Research in Medicine, Friedrich-Alexander Universität Erlangen-Nürnberg, Erlangen, Germany

**Keywords:** dementia, usability, development, digiDEM, cognitive impairment, older adults, aging, mobile health, mHealth, design, feedback, screening, user centred, cognitive disorder, user-centered, mobile app

## Abstract

**Background:**

Dementia is a widespread syndrome that currently affects more than 55 million people worldwide. Digital screening instruments are one way to increase diagnosis rates. Developing an app for older adults presents several challenges, both technical and social. In order to make the app user-friendly, feedback from potential future end users is crucial during this development process.

**Objective:**

This study aimed to establish a user-centered design process for the development of digiDEM-SCREEN, a user-friendly app to support early identification of persons with slight symptoms of dementia.

**Methods:**

This research used qualitative and quantitative methods and involved 3 key stakeholder groups: the digiDEM research team, the software development team, and the target user group (older adults ≥65 years with and without cognitive impairments). The development of the screening app was based on an already existing and scientifically analyzed screening test (Self-Administered Tasks Uncovering Risk of Neurodegeneration; SATURN). An initial prototype was developed based on the recommendations for mobile health apps and the teams’ experiences. The prototype was tested in several iterations by various end users and continuously improved. The app’s usability was evaluated using the System Usability Scale (SUS), and verbal feedback by the end users was obtained using the think-aloud method.

**Results:**

The translation process during test development took linguistic and cultural aspects into account. The texts were also adapted to the German-speaking context. Additional instructions were developed and supplemented. The test was administered using different randomization options to minimize learning effects. digiDEM-SCREEN was developed as a tablet and smartphone app. In the first focus group discussion, the developers identified and corrected the most significant criticism in the next version. Based on the iterative improvement process, only minor issues needed to be addressed after the final focus group discussion. The SUS score increased with each version (score of 72.5 for V1 vs 82.4 for V2), while the verbal feedback from end users also improved.

**Conclusions:**

The development of digiDEM-SCREEN serves as an excellent example of the importance of involving experts and potential end users in the design and development process of health apps. Close collaboration with end users leads to products that not only meet current standards but also address the actual needs and expectations of users. This is also a crucial step toward promoting broader adoption of such digital tools. This research highlights the significance of a user-centered design approach, allowing content, text, and design to be optimally tailored to the needs of the target audience. From these findings, it can be concluded that future projects in the field of health apps would also benefit from a similar approach.

## Introduction

Dementia is a widespread syndrome that currently affects over 55 million people worldwide, with annually almost 10 million new cases. The diagnosis of and treatment for people with dementia are going to be among the biggest challenges for health care systems worldwide [1]. A study by Eichler et al [2] found that 60% of people living with dementia in Germany had no formal diagnosis. Another problem lies in the long diagnostic periods. In the Bavarian Dementia Survey (BayDem) study, Wolff et al [3] found that the median time between the first perceived symptoms and diagnosis in Bavaria was 16 months. As Barth et al [4] were able to show, rural areas are also particularly affected here due to a high difficulty in accessing the facilities needed to diagnose and treat patients with dementia.

Screening instruments are one way of improving the diagnosis rate. A study with 146 participants has shown that diagnoses could be increased by almost 50% through upstream cognitive screening [2]. Internet-based screening tools offer the additional advantage that they can be used at a low threshold, regardless of time and place [5]. Digital technologies and the internet are already playing an increasingly central role in the everyday lives of older people. The proportion of people with internet access is growing across all age groups. In recent years, the number of German senior citizens (79‐84 years) who regularly use the internet has more than doubled (18.8% in 2011 vs 39.4% in 2017). There is also an increased interest in health websites among older people [6]. In the 2021 report published by the German Federal Office for Information Security, it was stated that around 163,000 different health apps existed [7]. However, there is a lack of high-quality dementia apps. As analyzed in an earlier study, for only 6 of 20 identified dementia apps, scientific evaluation studies have been published. In none of those studies, the effectiveness of the respective screening app could be proven. Among the published app evaluations, screening apps received the worst overall quality rating. In summary, the analysis showed that the existing apps at this time did not provide reliable information and results [8].

Thus, in the digiDEM Bayern project (Digital Dementia Registry Bavaria), we have not only focused on the establishment of a digital registry for persons with mild cognitive impairment and mild to moderate dementia [9,10], but also on the development, scientific evaluation, and sustained provisioning of innovative eHealth tools and digital apps [11,12]. The goal of our current project, in this context, was to establish a user-centered design process for the development of digiDEM-SCREEN, a user-friendly app to support the early identification of persons with slight symptoms of dementia. The objective of this publication is to illustrate the iterative and agile user-centered development process consisting of 8 phases to move from a conceptual idea to an early prototype and a final prototypical implementation with continual involvement and feedback process from stakeholders and intended future users.

## Methods

### Overview

In order to achieve the goal of a user-friendly screening app for people with slight dementia symptoms, a user-centered iterative development approach comprising the following steps was chosen:

1. A systematic literature research of scientifically evaluated digital and nondigital dementia screening tests.
2. An early prototype (V1) development based on the guidelines for graphic design and textual formulation criteria for people with cognitive impairments. Graphical requirements include an easy-to-understand layout, standardized navigation elements, and a clear division of instructions into several steps [13]. In addition, textual guidelines such as short and concise sentences, logically structured sections with headings and an active approach to the user should be observed [14]. Furthermore, the expertise of 3 clinicians from different disciplines with long-term experience in dementia research and 2 professors of medical informatics with expertise in developing mobile health apps, supported by their teams was incorporated into the development.
3. Conduction of an initial evaluation of the early prototype (V1) based on a focus group discussion (FGD) with potential end users (older adults ≥65 years with and without subjective cognitive impairments) [15]. The group discussion was recorded and transcribed afterward. The results were then categorized and analyzed based on a previously published qualitative content analysis [16]. The following categories were extracted: general linguistic adaptations, task-related linguistic adaptations, menu navigation, general navigation, and specific design changes to individual components.

1. Incorporating the FGD feedback and results into the specification for the prototypical implementation of digiDEM-SCREEN (V2).
2. The second evaluation with a new group of potential users (older adults ≥65 years with and without subjective cognitive impairments) was based on the think-aloud method, where participants speak their thoughts and wishes aloud during the test and are observed by a researcher who also takes notes [17].
3. Incorporating the user feedback and think-aloud evaluation results into the enhanced specification for the subsequent digiDEM-SCREEN development step (V3).
4. Conduction of an additional focus group evaluation of the improved beta version of the app (V3) with people with migration background (nonnative German speakers).
5. Development and deployment of the first ready-to-use digiDEM-SCREEN version (V4).

Recruitment of the facilities for participation (steps 4, 5, and 7) was based upon the network of research partners in the project digiDEM. The older adults from the facilities were informed about participation options in former group meetings (informed consent). After consenting, participants were invited to take part in the respective focus groups.

In steps 3 and 5, the System Usability Scale (SUS) has been calculated for the respective prototype versions by applying the German version of the standardized SUS questionnaire [18]. The scale can take values between 0 and 100; the higher the value, the higher the user-friendliness is categorized [19]. In addition, also in step 7, a self-assessment was used to determine technology use, interest, and expertise, each on a 5-point Likert scale (1 - ‘Does not apply at all’; 5 - ‘Applies completely’). The participants gave their subjective assessment and considered if they could use the app on their own (on a scale of 1 to 10) of the app and the specific components [20]. Furthermore, the participants were asked to name the most considerable problems associated with the app and if they wanted to change something.

Thus, our user-centered software design and development process included qualitative and quantitative evaluation methods at 3 different stages of the development process.

### Ethical Considerations

This study was approved by the Ethics Committee of the Medical Faculty of the Friedrich-Alexander Universität Erlangen-Nürnberg (application number: 20-253_1-B; August 14, 2023). Written consent was obtained prior to the user testing and focus group discussion. All participants data were pseudonymized. The list of reidentifying data was stored separately from the analyzed data, and only authorized individuals have access to them. No one was paid to test the app.

## Results

### Steps 1 and 2: Development of the First Prototype (V1)

Prior to developing the screening test, we conducted systematic literature research. The search terms are shown in Multimedia Appendix 1. Criteria for the decision on a suitable screening tool were the scientifically examined psychometric properties (sensitivity and specificity), the availability of being a free to use app (not paid), and the technical feasibility of a tablet/smartphone. The main source of information was the systematic reviews of Chan et al [21] as well as the specific studies of the screening tools [22,23]. The decision on the Self-Administered Tasks Uncovering Risk of Neurodegeneration (SATURN) was based upon a group discussion about the aforementioned criteria as well as the (methodological) quality of the screening tools and the underlying scientific studies in general.

The SATURN [24] proved to be a test with particular promising diagnostic values (sensitivity: 0.92; specificity: 0.88 in dementia cases vs controls) [21]. The test is usable via a tablet. Administration time is about 10 minutes, which can be especially beneficial for older adults as shorter tests might induce less fatigue and therefore be more suitable for repeat administration compared with lengthier instruments [25]. Thus, the SATURN provides the foundation for the development and validation of a German adaption of the test usable as an app via smartphone and tablet.

To date, there is no German version of the SATURN test. The translation of the English version of the SATURN into German was carried out independently by 2 research assistants from the digiDEM Bayern project (MZ and ND) using the translate-retranslate method. Apart from some general adaptations, such as the correct assignment of the users’ residence, linguistic aspects were also taken into account, and the texts were adapted to the German-speaking context. In some translations, the number of letters in the word increased noticeably (eg, farm - Bauernhof). A shorter related word (field - Feld) was then used in these places. Additional instructions were developed. The test adaptations aimed to ensure that both the implementation and the evaluation could be carried out entirely by the user or the system alone. At the start of the original SATURN test, the participant was asked to read aloud the task (close your eyes) and perform it [24]. Without a handler to check the action, there could be no subsequent evaluation (What phrase did you first read from this tablet?). Therefore, the researcher chose an alternative task (tap on the yellow circle) that also involved reading and performing an action.

The final screening test consists of tasks from 6 different cognitive domains: Comprehension, Visuospatial, Orientation, Memory, Calculation, and Executive Function. Points are awarded for each task, which adds up to a maximum score of 30. The tasks must be completed without the help of other people. Participants may use their visual aids to complete the tasks; all other aids (eg, paper and pencil) are not permitted. A detailed description and illustrations of the individual test tasks can be found in Multimedia Appendix 1.

**Figure 1.**
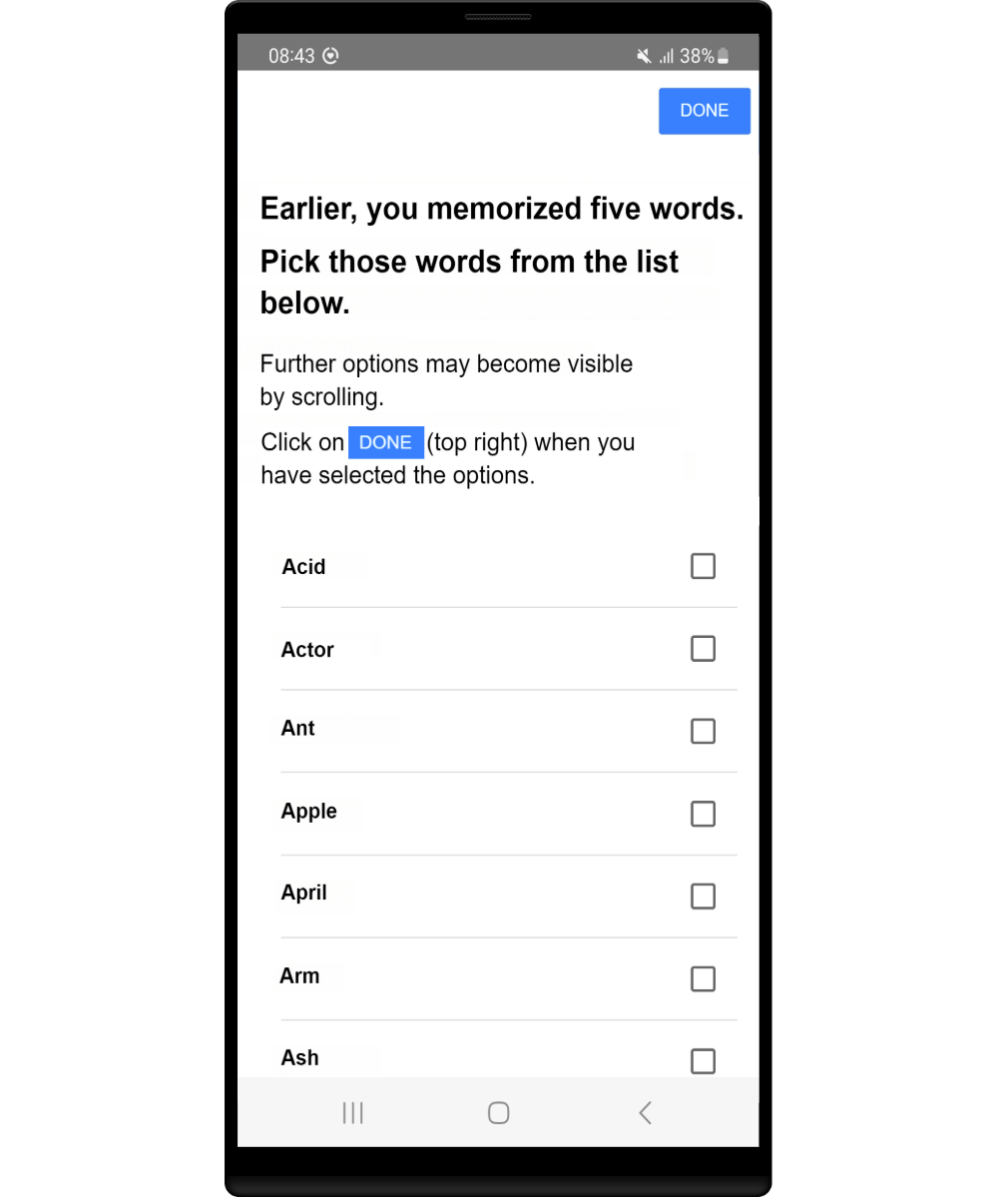
Word selection task as visible on a smartphone.

**Figure 2.**
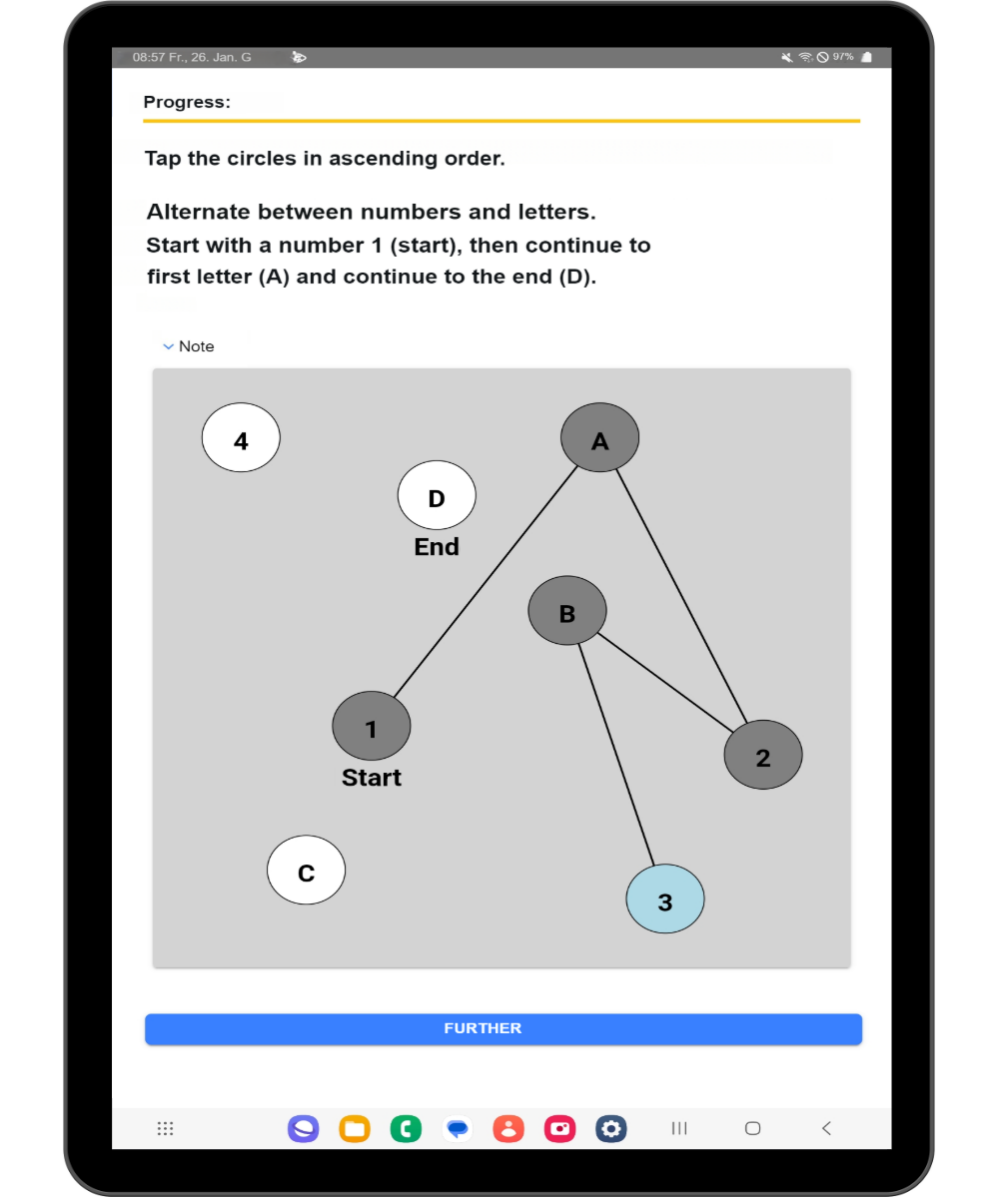
Trail Making Test as visible on a tablet.

Another innovation is that the authors are developing the app as a tablet and a smartphone version. Due to the smaller display sizes, new components like the word selection task shown in Figure 1 had to be created. The researcher also developed some new logic to prevent larger adjustments, for example, that the user is only allowed to undo the last connection at the last task (tap on the circle with a blue background; Figure 2).

The following table (Table 1) shows the baseline characteristics of the participants in the usability analysis. This is followed by a description of the details of the individual events.

**Table 1.** Baseline characteristics.

Study sample characteristics	FGD^a^ 1 (Prototype V1)		Usability test (Prototype V2)		FGD 2 (Prototype V3)	
Study population	13		21		7	
Age (years), mean (range)	75.8 (66-84)		70.2 (65-80)		60.8 (53-69)	
Sex, n (%)						
Male	1 (7.7)		8 (38.1)		0 (0.0)	
Female	12 (92.3)		13 (61.9)		7 (100.0)	
Education, n (%)						
Low	4 (30.8)		0 (0.0)		1 (14.3)	
Medium	5 (38.4)		10 (47.6)		1 (14.3)	
High	4 (30.8)		11 (52.4)		5 (71.4)	
Self-perceived cognitive impairment, n (%)	5 (38.4)		5 (23.8)		0 (0.0)	
Nonnative German speaker, n (%)	0 (0.0)		2 (9.5)		7 (100.0)	
SUS^b^ (0‐100), mean (SD)	72.5 (1.6)		82.4 (16.1)		—^c^	
App rating (1-10), mean (SD)	7.3 (2.1)		8.7 (1.5)		8.7 (1.2)	
Independent app use (1-10), mean (SD)	7.5 (2.9)		8.95 (1.3)		8.5 (1.0)	

a FGD: focus group discussion.

b SUS: System Usability Scale.

c Not applicable.

In both the first 2 focus groups, the SUS score was slightly lower in people with subjective cognitive impairment (FGD1: healthy older adults=74.4; people with subjective cognitive impairment=69.5; FGD2: healthy older adults=83.5; people with subjective cognitive impairment=82).

The SUS score decreased with advanced age (FDG1: ≥80 years old=63.8, 79‐70 years old=73.9, ≤70 years old=85.0; FDG2:≥80 years old=75.0, 79‐70 years old=77.5, ≤70 years old=85.0). People with a medium-level education had the best scores on the SUS (FDG1=85.0; FDG2=83.3), followed by people with a high-level education (FDG1=73.8; FDG2=83.0) and people with a low-level education (FDG1=55.6).

### Steps 3 and 4: Focus Group Discussion V1

The first FGD took place as part of a memory training group. A memory training group is a frequent meeting of older adults, in which those adults perform different memory training exercises under the supervision of a group leader. Frequent excursions are also part of this service. The service is offered by a nonprofit organization (German: Wohlfahrtsverband) and is led by a research associate in the project digiDEM. The group consisted of a total of 13 participants. Their baseline characteristics are shown in Table 1. On average, they used modern technologies frequently (3.39) and showed an average interest in technological innovations (3.00). Their self-assessment of their competence in using modern technology was moderate (2.39), but the fear of failure played only a moderately important role (2.69).

Due to the large number of participants, 3 small groups were formed for the test. Participants were able to extensively test the app prototype and contact a research assistant with any questions. The individual components of the prototype achieved a subjective app rating of 7.3 (out of 10) points and an SUS score of 72.5. Participants also generally felt able (7.5 out of 10) to use the app independently without outside help. Subsequent group discussion of the results took place again in a large group.

**Figure 3.**
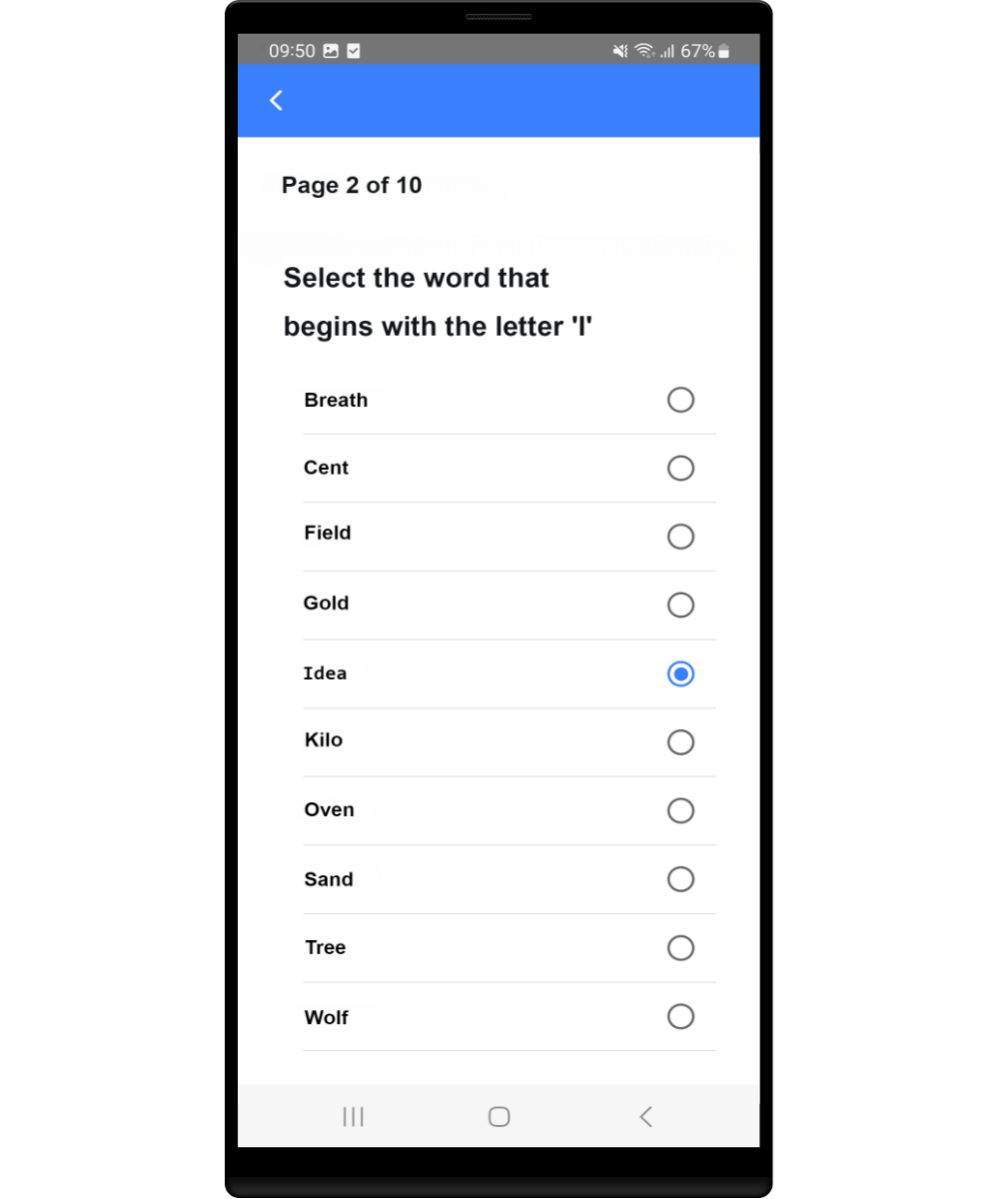
Illustration of the prototype (**V1**).

**Figure 4.**
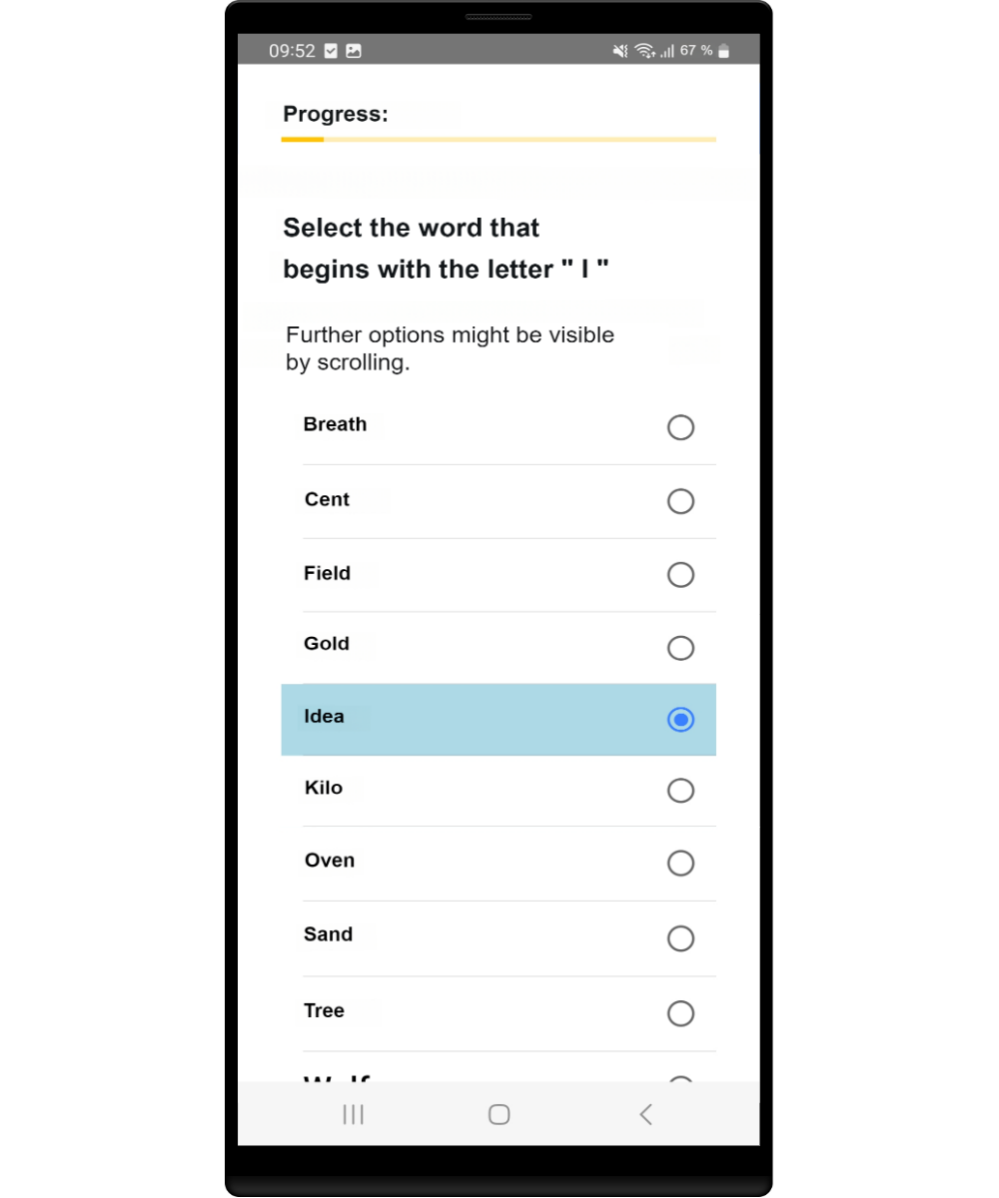
Final visualization (**V4**).

The 2 most significant areas of improvement were observed in all the 3 small groups. Many participants recognized the letter I as T due to the inverted commas (‘I’) and had problems answering this task correctly. Participants also did not always recognize the selected word, as only the radio button on the right-hand side of the prototype showed their selection. The app prototype (V1) is shown in Figure 3. Participants therefore specifically requested that the entire line be colored when a selection was made. The 2 adjustments are shown in Figure 4.

In addition, minor inconsistencies were noticed in this test, such as the fact that sometimes “Next” and sometimes “Done” were used to move on to the next task. The participants also wanted the selected images to be marked more clearly and the contrast and color intensity to be adjusted so that the colors could be recognized more clearly. One participant commented that she liked the “simple design” and that it did not distract from the actual content. Another participant mentioned that the instructions were too complex (“They were good instructions that you could actually understand. But I really had to read very carefully”). Therefore, the descriptive text has been simplified. Feedback on the user-friendliness of the FGD was predominantly positive. One participant particularly liked the fact that she could use the app without having much prior knowledge. The general consensus was that the app was easier to use on a tablet than on a smartphone due to the larger screen size. However, the smartphone version was also rated as usable by participants. These points mentioned were discussed with the developers and incorporated into the second prototype accordingly.

### Steps 5 and 6: Think-Aloud Usability Evaluation (V2)

A total of 21 older adults, who were randomly selected from participants in a dementia prevention event for older adults (≥65 years), participated in this usability test. Their baseline characteristics are shown in Table 1. They most frequently use modern technology (4) and are interested in technical innovations (3.62). They rated their competence in modern technology as average (3.33), while the fear of failure did not play a significant role (2.48).

The quantitative key figures collected increased compared with the first version. This prototype achieved a subjective app rating of 8.7 (out of 10) points and an SUS score of 82.4. The participants’ assessment of using the app independently, without external help, also increased significantly (8.95 out of 10).

Based on the researchers’ observations and the participants’ statements minor adjustments and precisions, such as allowing €67 and €67.00 as the correct answer in the calculating task, were made. Some users also commented negatively about the last task’s descriptive text. Due to the length and complexity of the content, the question was often not solved or only solved with a hint from the research assistant. Based on this feedback, the language of the text was revised again.

### Step 7: Focus Group Discussion With Nonnative Speaker (V3)

The last user test took place under the aspect of accessible language and comprehensibility. To this end, an FGD was conducted with people with a migration background. Seven older adults took part in this FGD. Their baseline characteristics are shown in Table 1. They came from 4 different countries (Iraq, Kuwait, Sri Lanka, and Syria) and were all nonnative German speakers. They most frequently use modern technology (3.86) and are interested in technical innovations (3.57). They rated their competence in modern technology as average (3.14), while fear of failure played a minor role (2.58).

The quantitative indicators collected were similar to those of the German-speaking users. This prototype achieved a subjective app rating of 8.7 (out of 10) points. These participants also rated the success of using the app independently, without external help, at 8.5 (out of 10). Unfortunately, no SUS score could be obtained from this group due to the language barrier.

Two relevant changes emerged from the group discussion. First, a note on scrolling (Figure 4) was added in the appropriate places, and second, the language was adapted. A total of 5 of the 7 participants answered one of the initial questions (Select the fruit from the list.) incorrectly. The participants confused 2 words “Kirsche (cherry)” and “Kirche (church),” which are very similar in German. As this error does not indicate a possible cognitive decline, the word “Kirche (church)” was changed to “Kapelle (chapel).”

### Step 8: Development and Deployment of the First Ready-to-Use digiDEM-SCREEN Version (V4)

In this step, the digiDEM-SCREEN test was finalized as a screening app for recording the current cognitive status of users. A validation study is currently underway. The test will be administered to patients in outpatient memory clinics and its sensitivity and specificity will be evaluated in the context of existing diagnoses and other nondigital cognitive tests. As part of the validation, cut-off values for categorizing current cognitive ability will also be determined as part of the validation. Depending on the test result, the user is given a short recommendation and options for action. If the result is above the threshold value calculated in the validation study, the screening does not indicate memory impairment. It is recommended that the test be repeated at regular intervals to monitor changes in memory performance. If the final result is below the threshold, further neuropsychological assessment in a memory outpatient clinic is recommended.

A study is currently underway to determine the sensitivity and specificity of the developed screening test (V4) and its correlation with the Montreal Cognitive Assessment [26]. For increased transparency, the research group has registered the project in the German Clinical Trials Register (DRKS) (registration number: DRKS00033764). After validation, the test will be available free of charge to anyone interested. Different randomization options have been used to minimize the learning effect. There are 5 versions of the test, which differ in the order of the numbers to be memorized. In addition, the position of each answer option is randomized for each test session. There are also plans to offer the screening test in different languages in the future. The possibility of multilingualism has already been taken into account in the programming of the app. This will be easy to implement once further translations have been validated.

## Discussion

### Principal Findings

There is a lack of evidence in the field of freely accessible apps for people with dementia, especially screening apps. A study published in 2023 showed that there are not any scientific studies to prove the effectiveness of any of the German-speaking screening apps [8].

A user-friendly app should have 3 main characteristics: typography appropriate for the target group (eg, recognizable icons), intuitive operation, such as fewer clicks to the desired action, and simplicity (eg, simple navigation) [27]. Three key stakeholder groups were involved in developing the digiDEM-SCREEN app: the digiDEM research team, the software development team, and the target user group (older adults ≥65 years with and without subjective cognitive impairments). Developing an app for older adults presents several challenges, both technical and social. Older adults may have less technology experience and difficulty understanding complex user interfaces [28]. Many older adults also have age-related limitations, such as visual or hearing impairments [29]. Cognitive abilities can decline with age, making complex apps more difficult to use. The app should be tailored to the cognitive needs of older users, for example, by providing clear instructions and simple interactions. Considering these challenges when developing an app for older adults can help to create a user-friendly and accessible application that improves the lives of older people and promotes their independence [27]. In order to make the app as user-friendly as possible, feedback from potential future end users is essential. A critical examination of the study population shows that the participant structure is dominated by women in terms of gender. This could lead to a distortion of the results, as the findings may not be transferable to the entire target group. However, an empirical comparison of the usability of a mail app between male and female users showed that there are no statistically significant differences in the performance criteria of efficiency, effectiveness, and satisfaction between the 2 groups [30]. Another study examined whether there were systematic differences between women and men in the evaluation of the user experience of 3 websites and showed that there were no significant differences between the genders. Personal attitudes and preferences have a greater influence on the results [31].

The activities summarized under the term “patient and public involvement” enable patients to be actively involved in the planning and development of new products. International associations such as Alzheimer Europe as well as scientists are very interested in encouraging the active involvement of people with dementia in research for brainstorming and counseling [32].

Digital health apps often struggle with low adherence. One possible reason for this is users’ personal frustration with the content of the intervention, the way it is presented, and the nonintuitive handling [33]. The selected user-centered design could sustainably increase adherence. Users have unique knowledge, perspectives, and experiences that can influence a product’s quality, appropriateness, and user-friendliness. User testing is an essential part of the iterative development process and contributes to increasing the quality and success of the app [34]. Prototypes make it possible to recognize potential problems or weaknesses in user interaction or design at an early stage. By discovering these problems at an early stage, expensive changes or new developments in later phases of development can be avoided [35]. Thus, using the prototype design in the first workshop provided the team with a cost-effective way to get feedback and evaluate the idea. In the user tests, the tablet prototype of the app performed better than the smartphone version. The participants mainly criticized the smaller font and display size, which made it somewhat difficult to enter answers in some places. Despite these criticisms, the smartphone version was still rated as user-friendly. Smartphones are the most common mobile devices. A study by Weber et al [36] from 2020 found that an average of 41.4% of 71.6-year-old participants used their own smartphone. Due to the high availability of smartphones among seniors, the research team decided to stick with both versions. The mobile app also works offline, so no internet connection is necessary. Those decisions made it possible to reach a larger number of potential users. During the test phases, the participants did not use their devices, which they were familiar with in everyday life, but devices provided by the research team. For example, the display size, operating system (or at least the version), and individual settings may differ from their device. It is expected that user-friendliness will be even higher when users utilize their own smartphone or tablet.

The workshop participants were positive about the experience and gave constructive comments on the app. In addition, the SUS score also increased with each iteration of the app version. Bangor et al [37] described that products with a SUS score of 90 points and above were rated as exceptional, products with a SUS score of 80 points were rated as good, and products with a SUS score of 70 points were rated as acceptable. Anything below 70 points had usability issues that were a cause for concern. That means all SUS assessments are above the average and at least rated as acceptable. The prototype V1 gained a SUS score of 72.5 from the participants of the FGD. The rating of the second app version was notably better (SUS score: 82.4) and, therefore, rated as good. Due to some comprehension difficulties, unfortunately, no SUS score could be raised in the second FGD. Whenever questionnaires are used directly with people with dementia, the questions should be short and understandable (no technical terms), and double negatives should be avoided [27].

The results of the user evaluations showed that a user-friendly screening test for people with subjective cognitive impairments could be developed for the German-speaking population.

The main focus of the focus groups was on minimizing potential sources of error. Nevertheless, there is still a residual risk that the test cannot be carried out properly. If the first 3 simple test tasks are not answered correctly, an end screen appears with the message that the test cannot be carried out due to technical or language barriers. In this case, the user is advised to visit a specialized clinician. In the general instructions before the start of the test, participants are also informed that for example visual aids should be used.

### Strengths

Different potential end users were included in the development process of the digital screening test as an app in order to improve usability and avoid technical or linguistic barriers. Moreover, additional languages and other extensions like a dementia prevention module can be added to the app.

### Limitations

There was no random sampling of participants. Furthermore, although the SUS scale is the most frequently used scale to assess the user-friendliness of IT applications, it also has its weaknesses. The results of a systematic review show that some studies found that the double-negative questions from the SUS are challenging to understand for people with dementia [27]. With this knowledge in mind, we used simple language and no double negatives for the remaining questions we phrased. In the first FGD, the researchers had to explain individual questions to the group, and no SUS score could be collected in the last FGD. Most of the participants needed help understanding the questions. They were, therefore, unable to give reliable answers.

### Conclusions

The development of digiDEM-SCREEN serves as an excellent example of the importance of involving experts and potential end users in the design and development process of health apps. From the initial stages of the project, experts were engaged in the content and design realization, providing a solid foundation for further development. The intensive testing phase, in which various end users tried out the app prototype in several iterations, clearly demonstrated the value of early and continuous feedback for improving the final product. This research highlights the significance of a user-centered design approach, allowing content, text, and design to be optimally tailored to the needs of the target audience. From these findings, it can be concluded that future projects in the field of health apps would also benefit from a similar approach. Research teams and app developers should integrate user-centered design practices into their development processes to ensure that the applications they create are not only functional but also user-friendly and appealing to the target audience. Such an approach could significantly enhance the acceptance and effectiveness of health apps, thereby making a valuable contribution to digital health care. A close collaboration with end users leads to products that not only meet current standards but also address the actual needs and expectations of users. This is a crucial step toward improving health technology and promoting broader adoption of such digital tools.

## Supplementary material

10.2196/65022 Multimedia Appendix 1 Search terms and detailed test description.

## Abbreviations

BayDem: Bavarian Dementia Survey
FGD: focus group discussion
SATURN: Self-Administered Tasks Uncovering Risk of Neurodegeneration
SUS: System Usability Scale
