# Supplementary material for: A User-Centered Design Approach for a Screening App for People With Cognitive Impairment (digiDEM-SCREEN): Development and Usability Study
Source: JMIR Hum Factors. 2025 Jan 22;12:e65022. doi: 10.2196/65022 (PMC11779685; doi:10.2196/65022)
Supplement: Multimedia Appendix 1 [file humanfactors-v12-e65022-s001.docx]

**Multimedia Appendix 1**

**Search terms:**

**(**‘remote’ **OR**

‘webbased’ **OR**

‘web-based’ **OR**

‘digital’ **OR**

‘virtual’ **OR**

‘online’ **OR**

**AND)**

‘dementia’ **OR**

‘alzheimer’

**AND**

‘screening ‘**OR**

‘dementia screening’ **OR**

‘cognitive screening’ **OR**

‘cognitive test’ **OR**

‘screening test’

**AND**

‘valid*’

**Detailed Test Description:**

The "Self-Administered Tasks Uncovering Risk of Neurodegeneration (SATURN)" was chosen as the basis for the development of a German-speaking screening test [22]. The translation of the English version of the SATURN into German was carried out independently by two research assistants from the digiDEM Bayern project (MZ and ND) using the translate-retranslate method. Apart from some general adaptations, such as the correct assignment of the users’ residence, also linguistic and cultural aspects were also taken into account, and the texts were adapted to the German-speaking context. Additional instructions were developed. The test adaptations aim to ensure that both the implementation and the evaluation can be carried out entirely by the user or the system alone.

The final screening test consists of tasks from 6 different cognitive domains: Comprehension, Visuospatial, Orientation, Memory, Calculation, and Executive Function. Points are awarded for each task, which adds up to a maximum score of 30. The tasks must be completed without the help of other people. Participants may use their visual aids to complete the tasks; all other aids (e.g., paper and pencil) are not permitted.

The language in the figures shown below is the original digiDEM-SCREEN language (German).

The test starts with a question ("Tap the yellow dot") to check whether participants understand and can perform a simple task (Figure 1). In the "Comprehension" section, participants are asked to (1) select the one word (out of 8) that begins with "I" (Figure 2), (2) select the two nouns (out of four) that represent fruit (Figure 3), and (3) enter the number shown using the numeric keypad on the screen (Figure 4). The "Visual Spatial" section shows a large picture and six smaller pictures. The participants must now select two of the six possibilities that would make up the large picture together (Figure 5). In the "Orientation" section, participants are asked to select the federal state they are in (from the alphabetically listed options) (Figure 6), the day of the week (from seven listed options) (Figure 7), the month (from twelve options) (Figure 8) and to enter the correct year using the numeric keypad (Figure 9). In the "Memory" section, participants are repeatedly asked to memorise the five words, which are then displayed one after the other in large font for 2 seconds per word with a 1 second break in between (Figure 10). We first test memory by asking participants to select the instruction they read at the beginning of the task from six options ("tap the yellow dot") (Figure 11). We ask them which word (from eight) they selected that corresponds to a particular letter (related to the "I" word) (Figure 12), and then we ask them which number they entered previously (Figure 13). Participants are then asked to select the five memorised words from 100 options (Figure 14, 15). The "Calculation" section comprises two related tasks that are intended to reflect an everyday situation in a supermarket (adding up the prices of two goods and calculating change) (Figure 16, 17). In the "Execution" section, participants must first select the colour word ("red", "green" or "blue") in which the respective word displayed is described (Figure 18). In the second part, participants must connect the circles correctly (Figure 19).


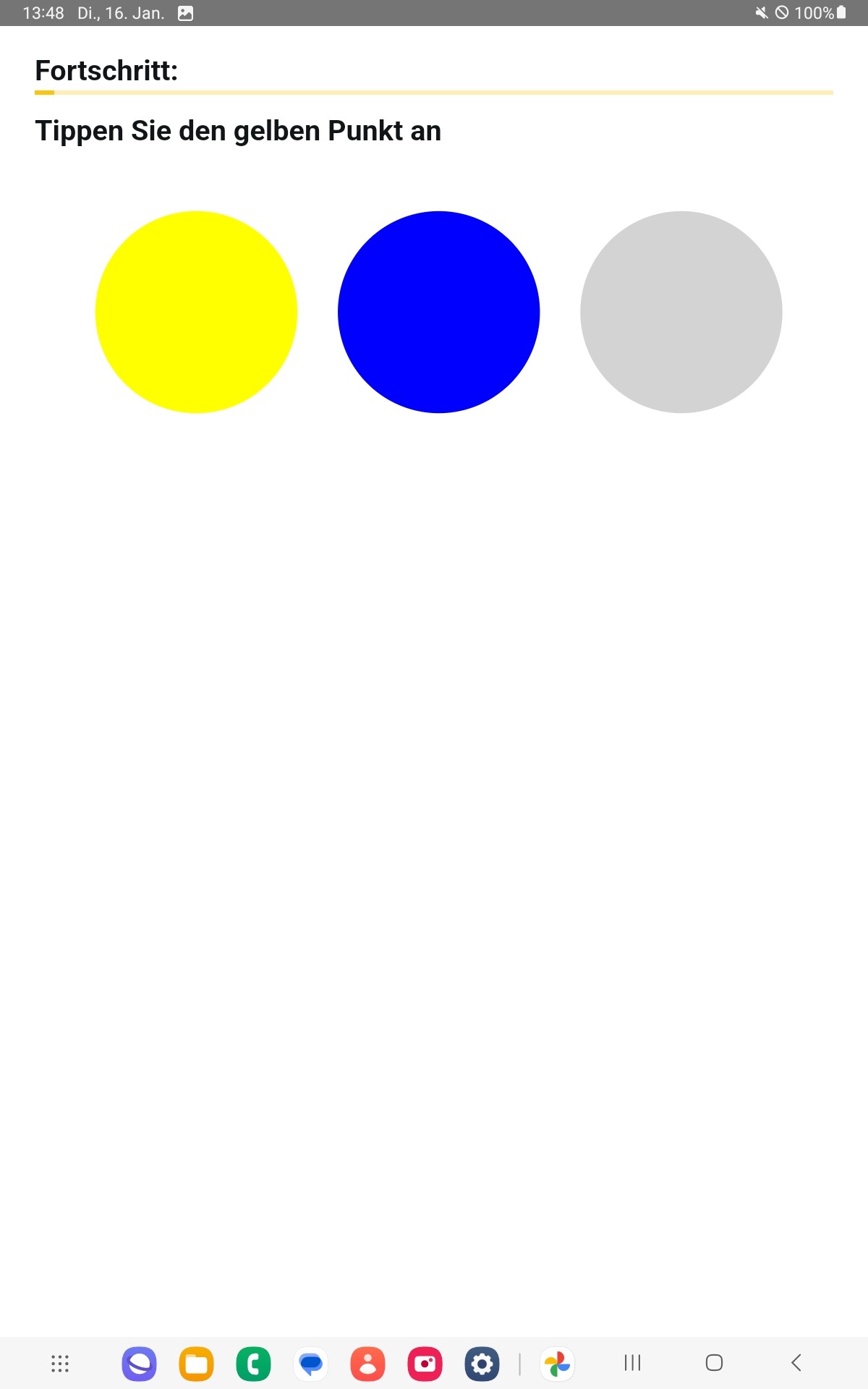


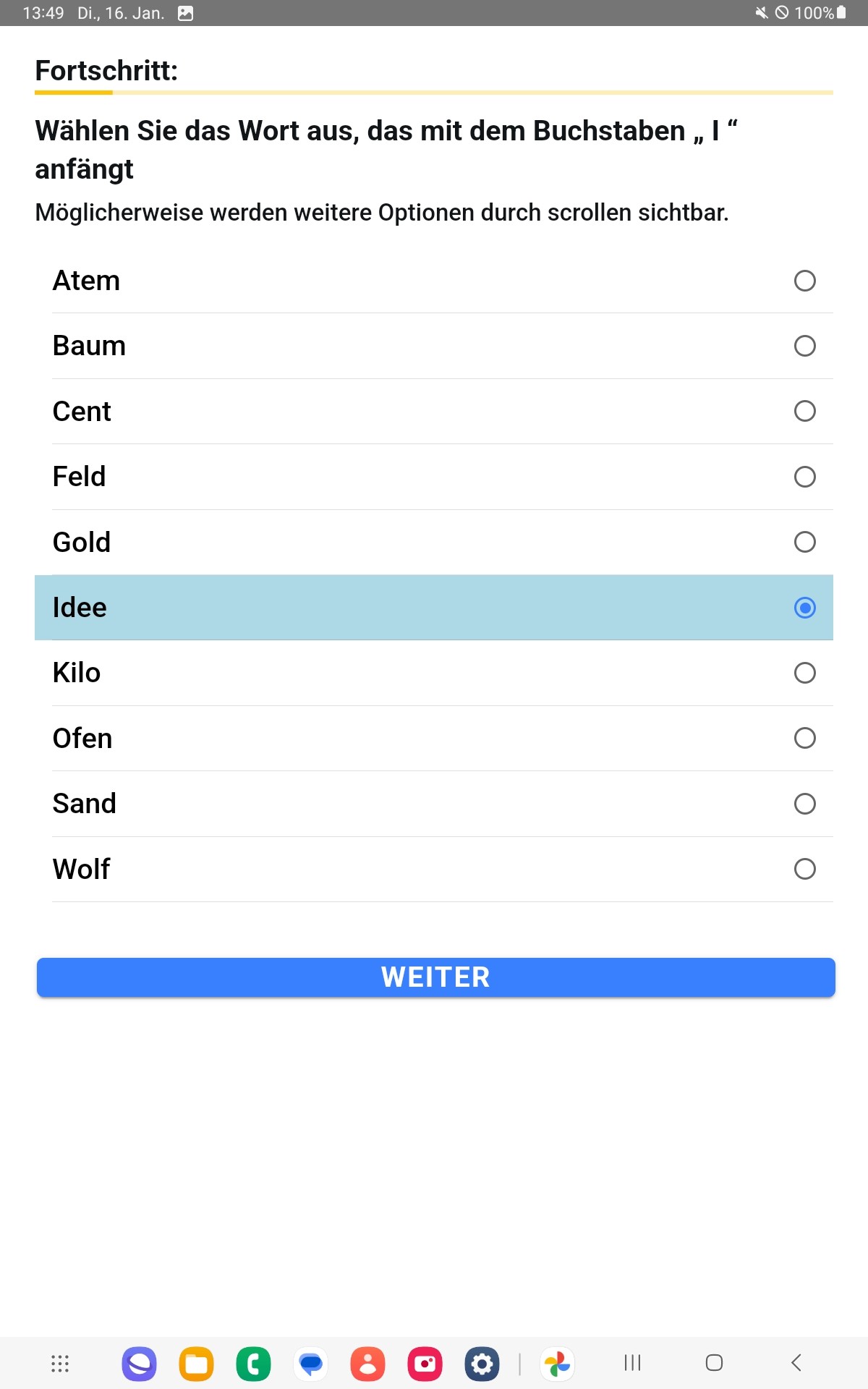


Figure 1: Entry-Task

Figure 2: Task 1 (Comprehension Section)


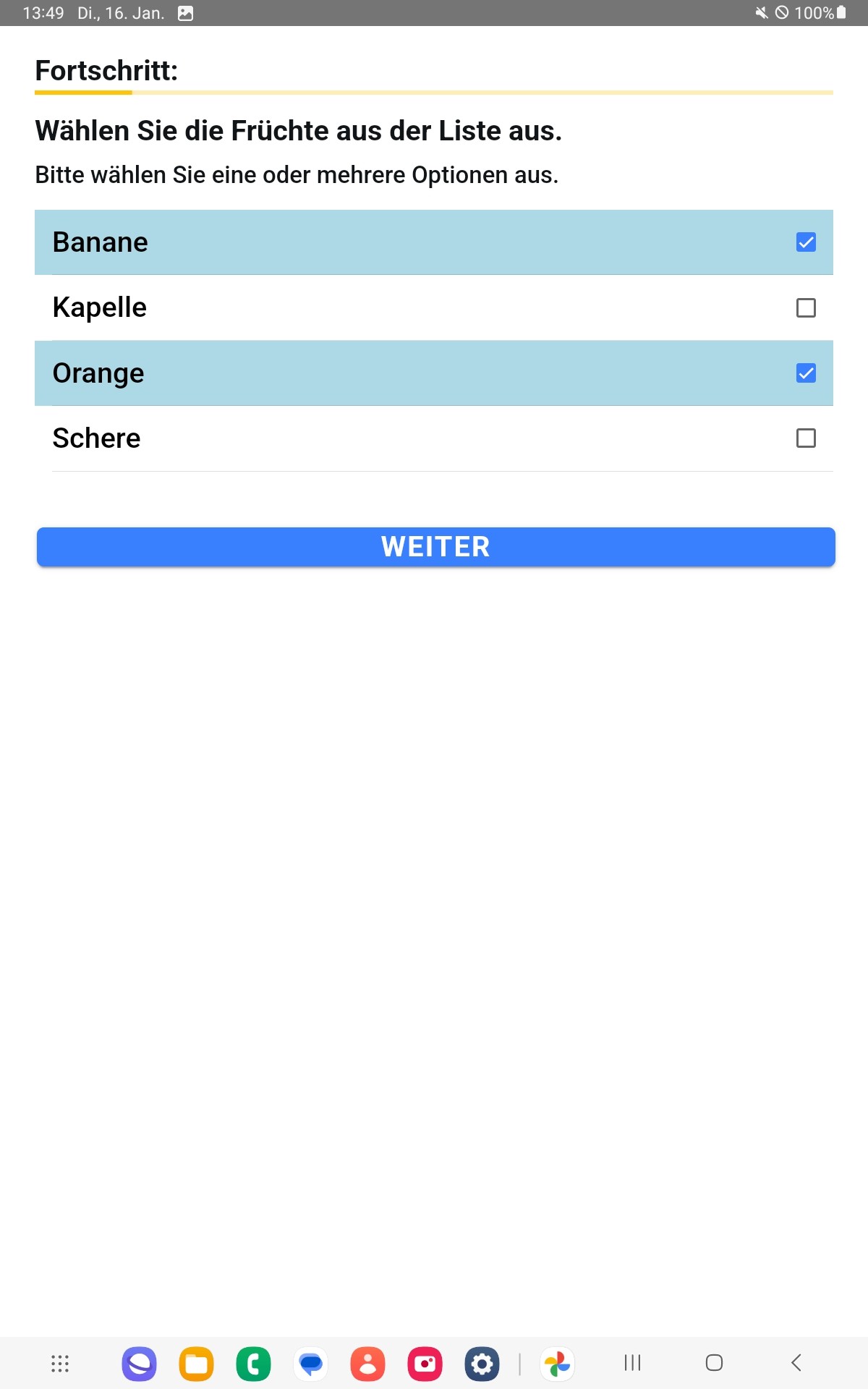


Figure 3: Task 2 (Comprehension Section)


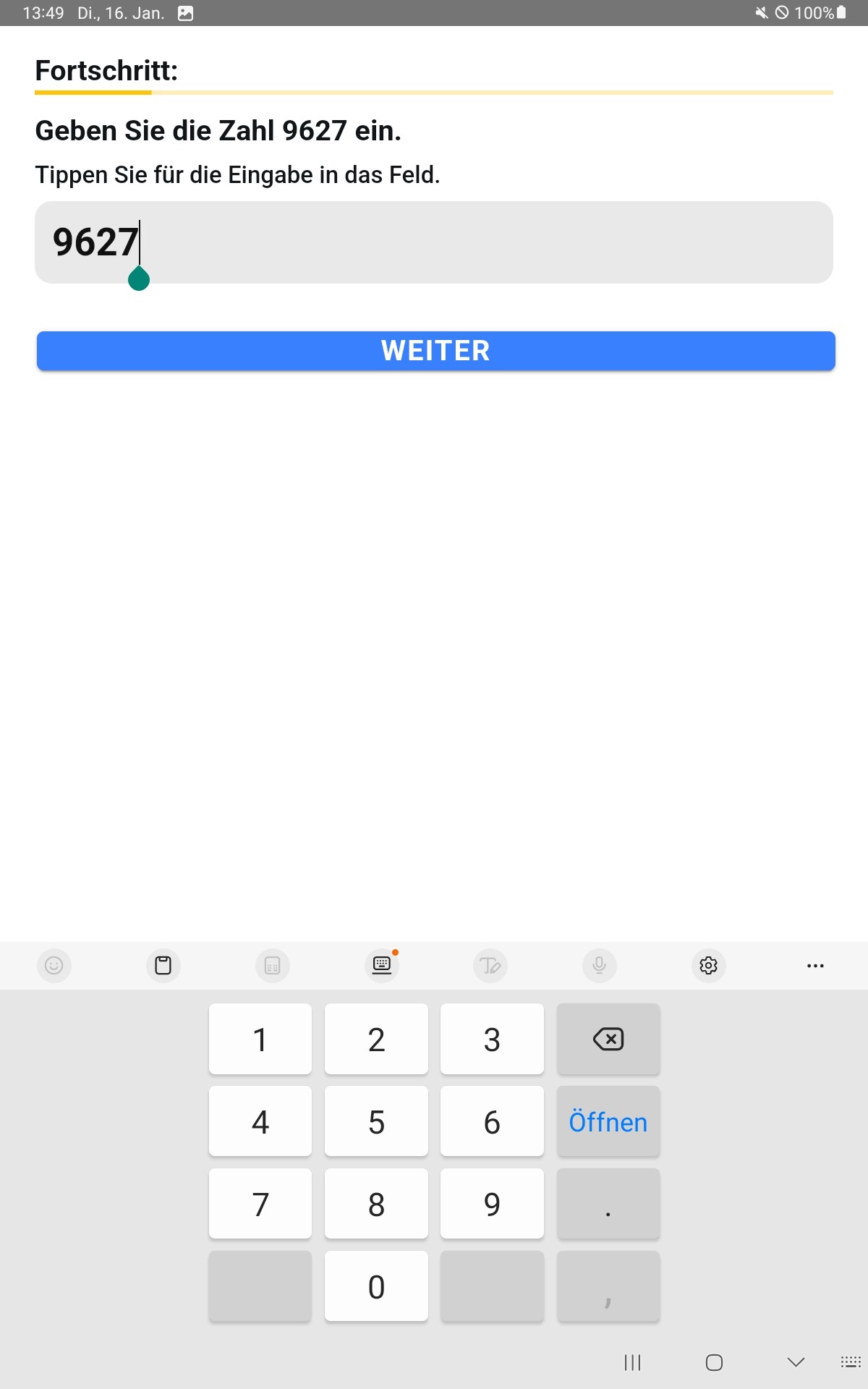


Figure 4: Task 3 (Comprehension Section)


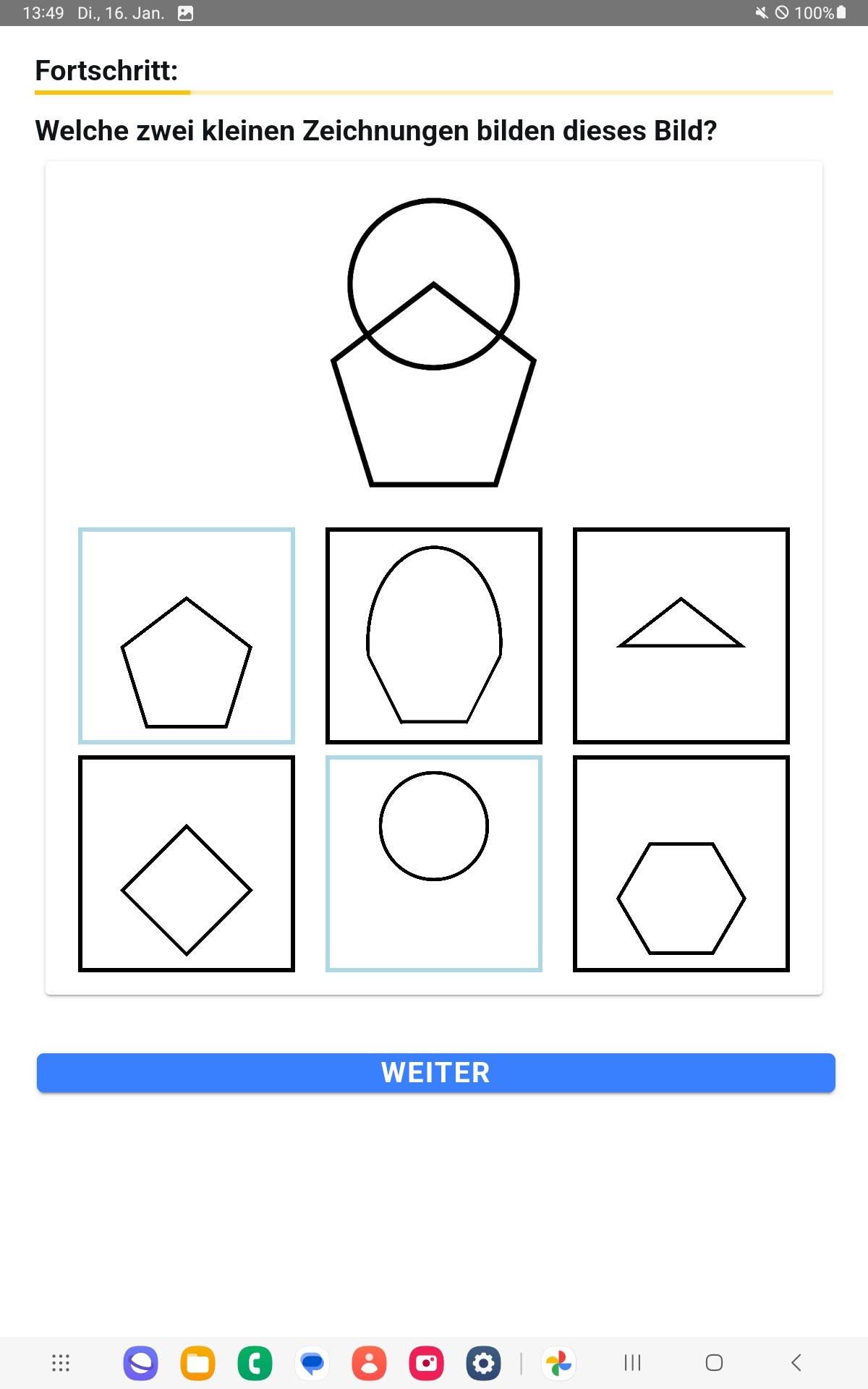


Figure 5: Task 4-7 (Visual Spatial Section)


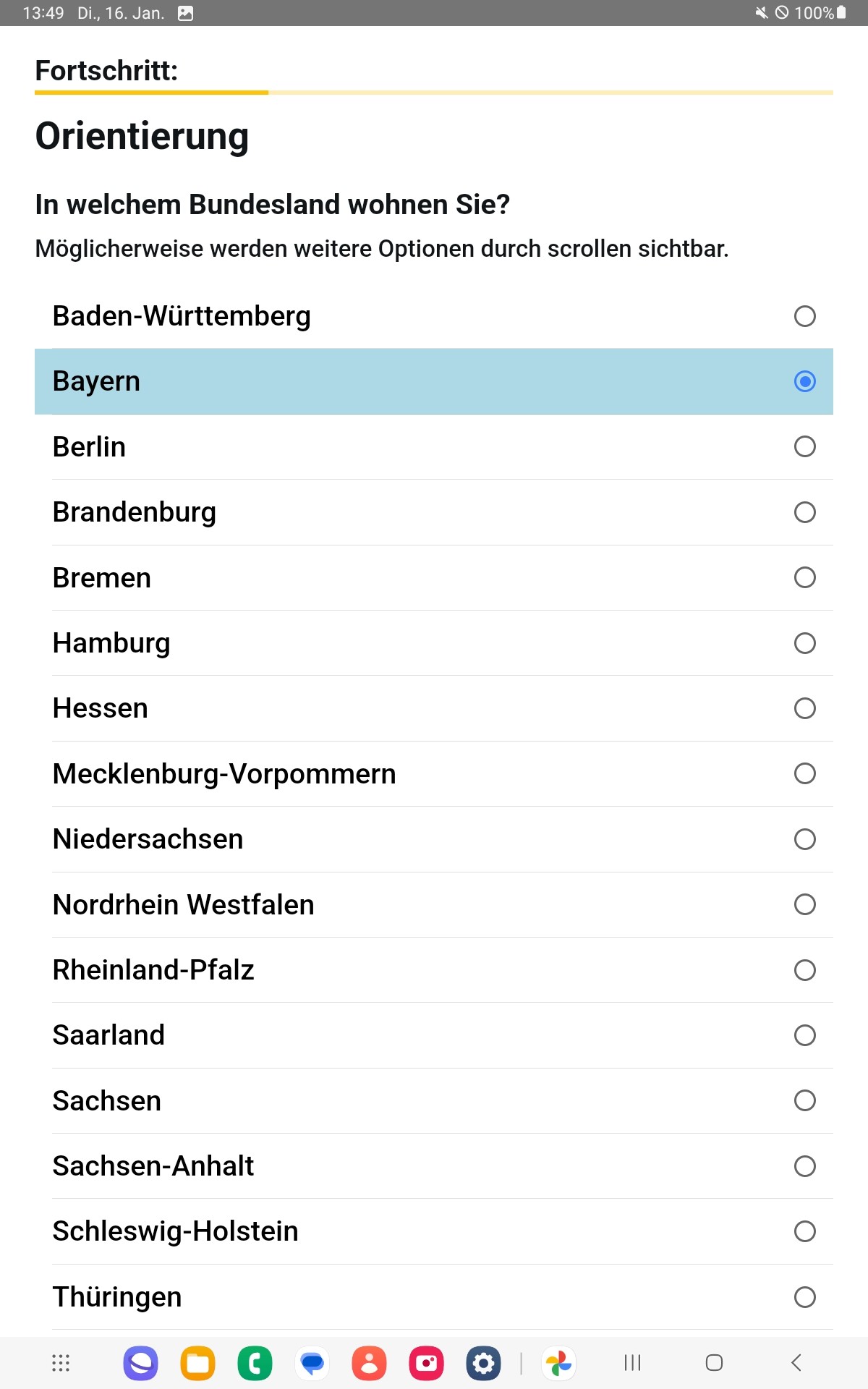


Figure 6: Task 8 (Orientation Section)


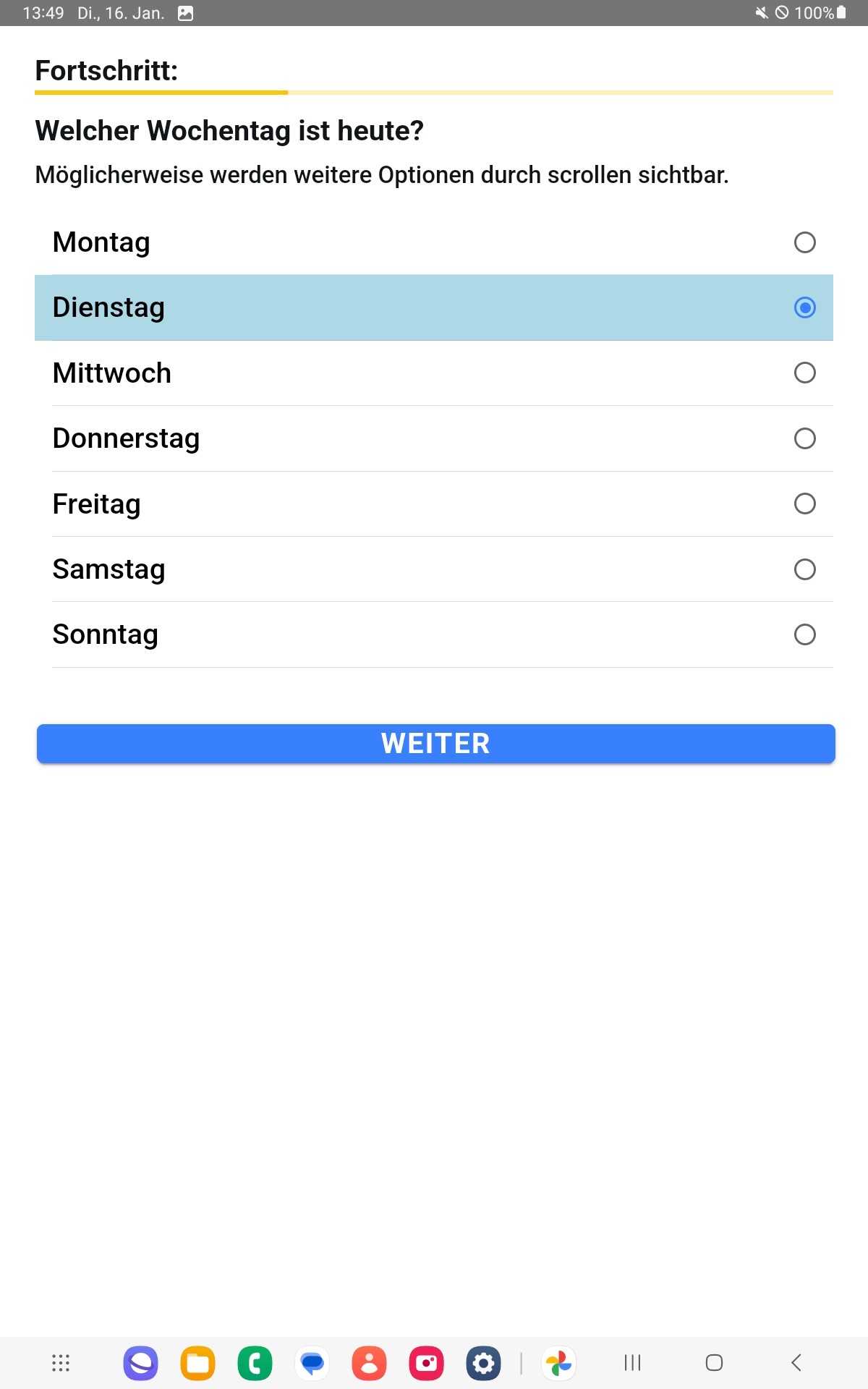


Figure 7: Task 9 (Orientation Section)


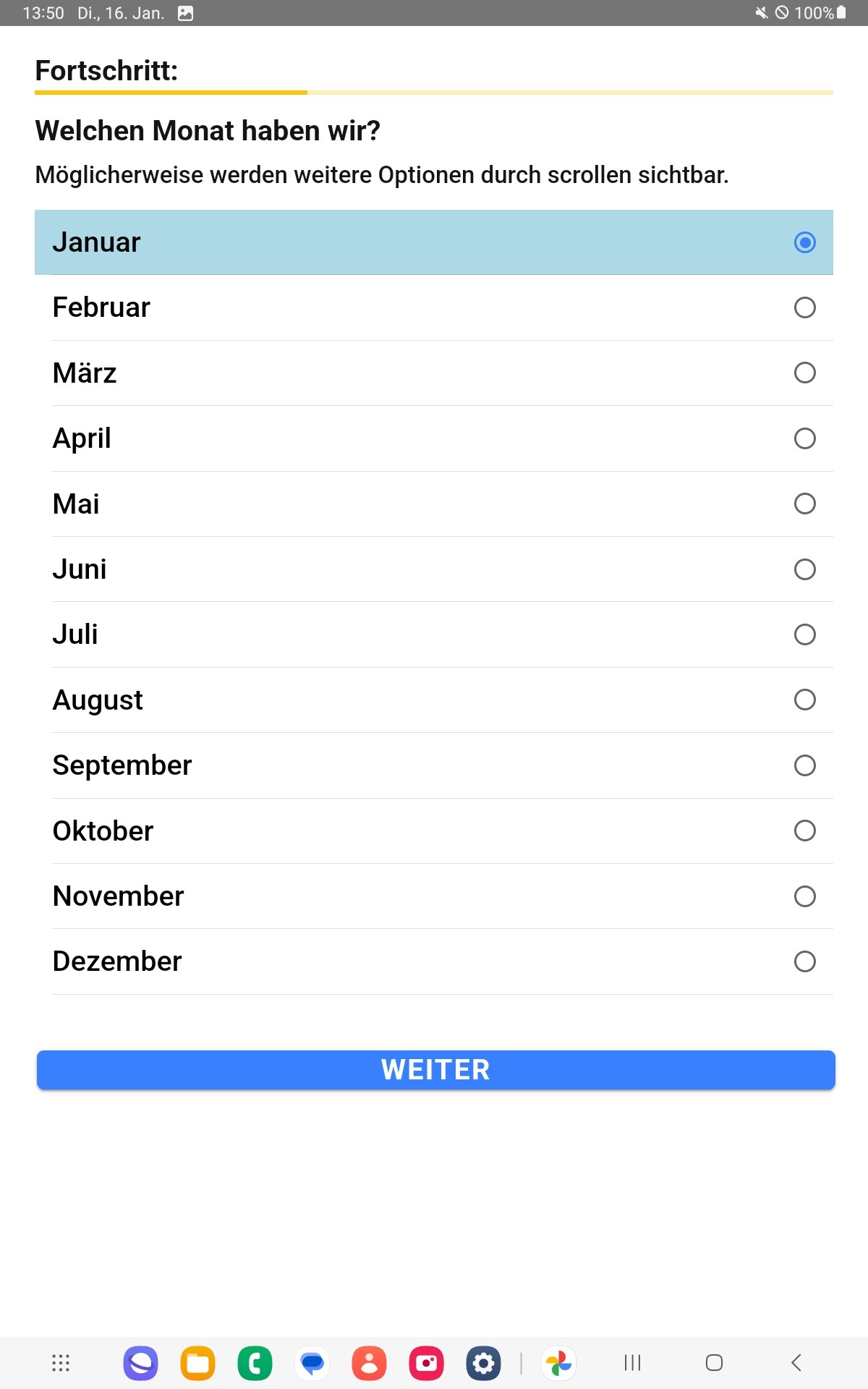


Figure 8: Task 10 (Orientation Section)


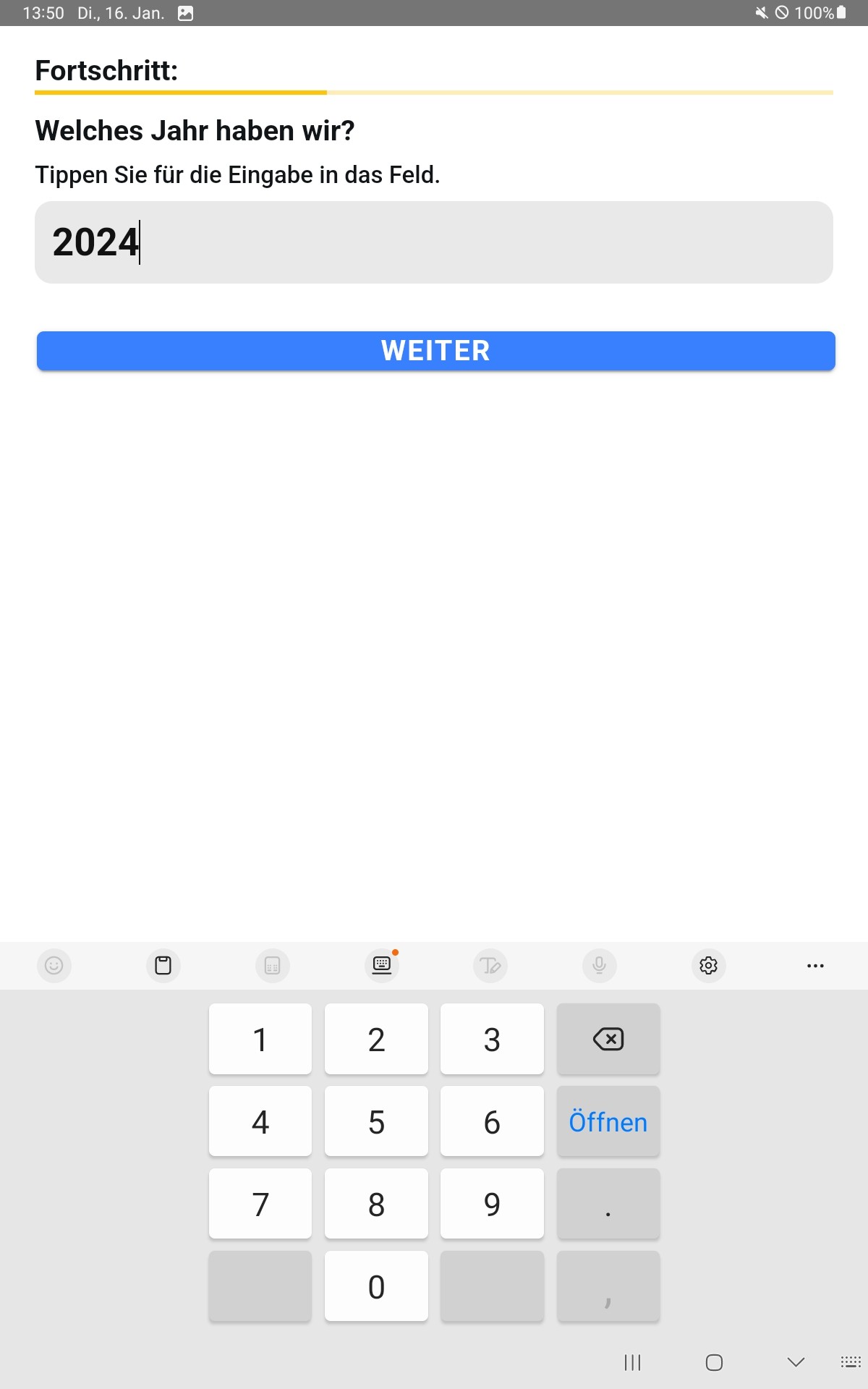


Figure 9: Task 11 (Orientation Section)


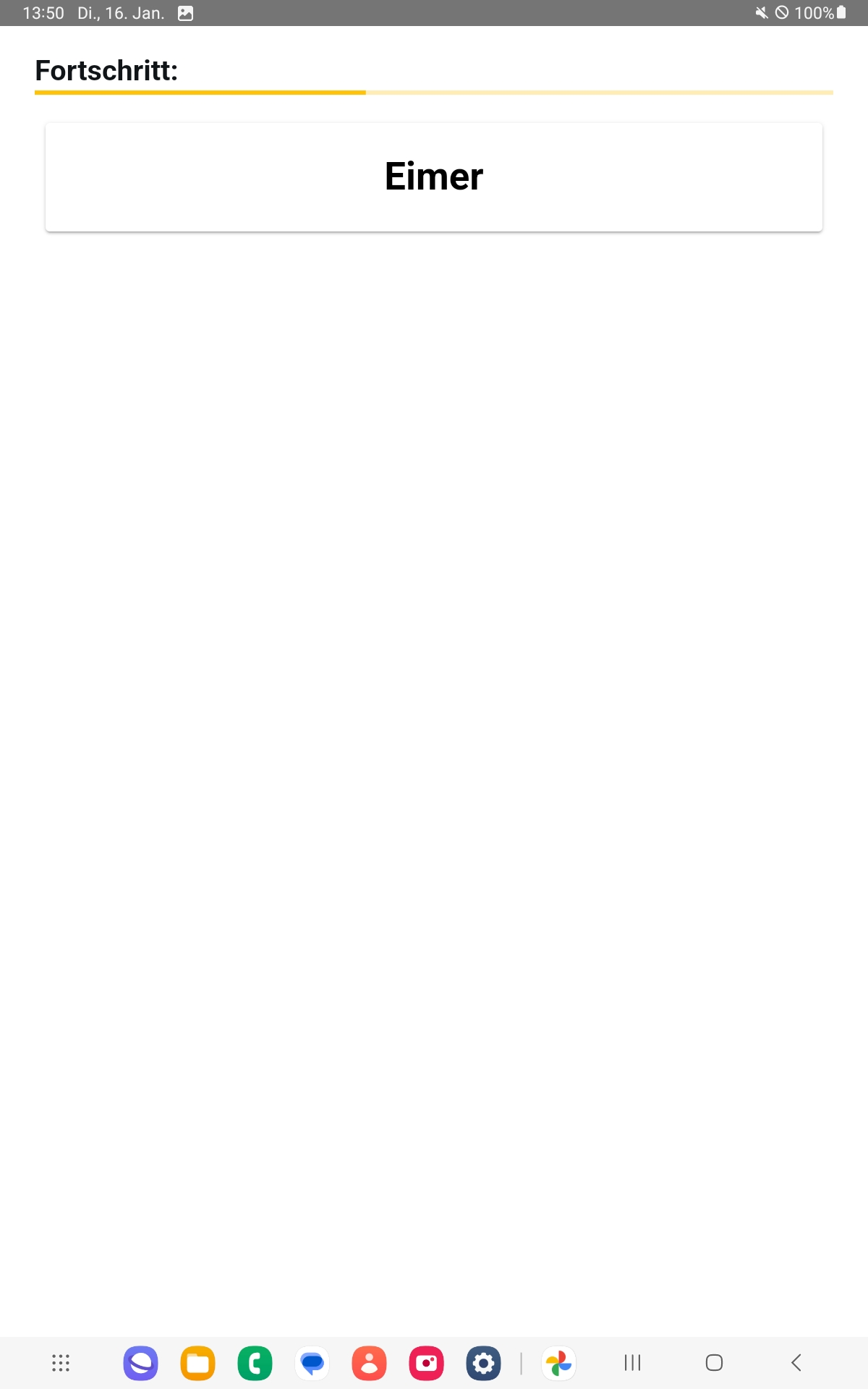


Figure 10: Task 12-16 (Memory Section)


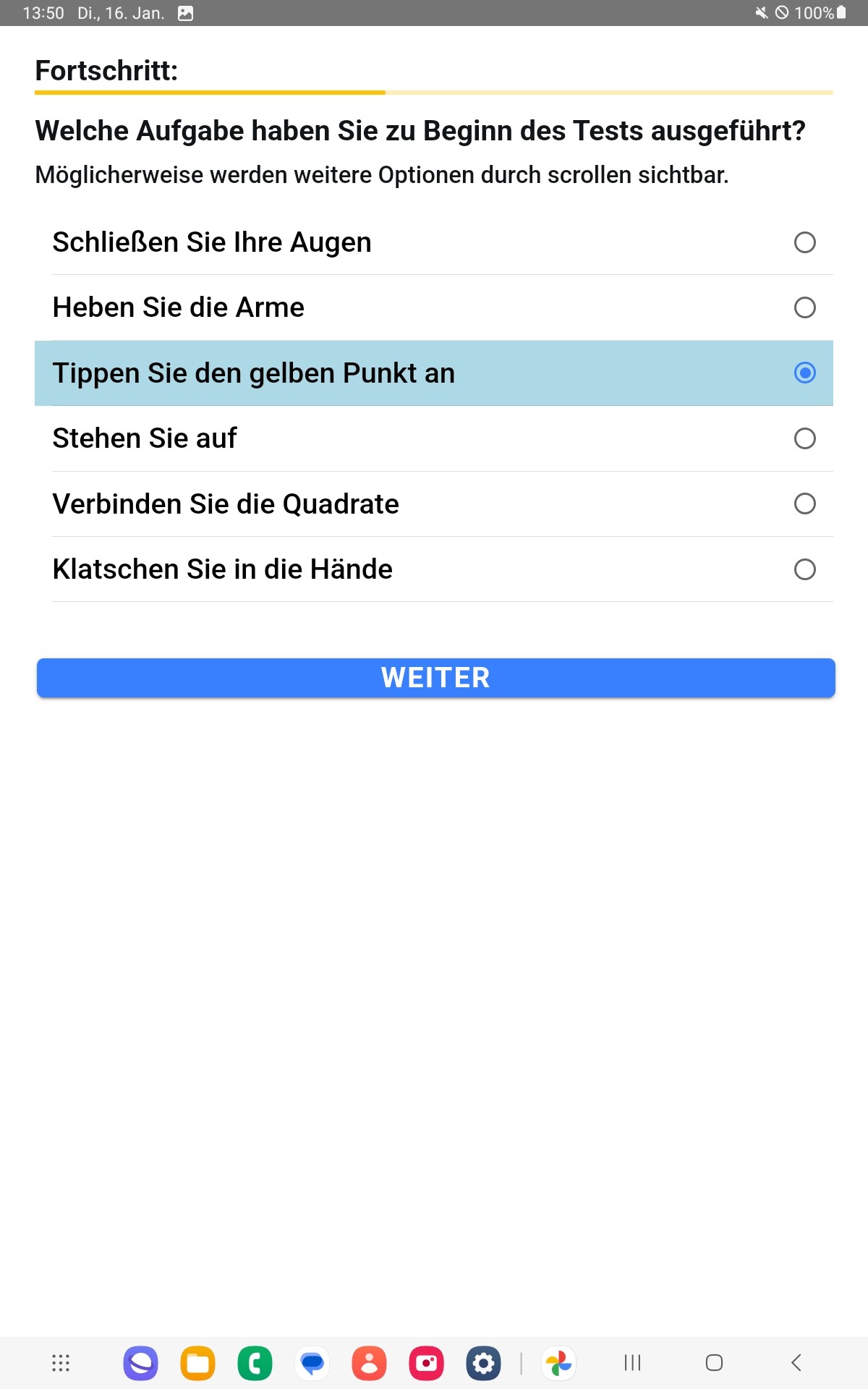


Figure 11: Task 17 (Memory Section)


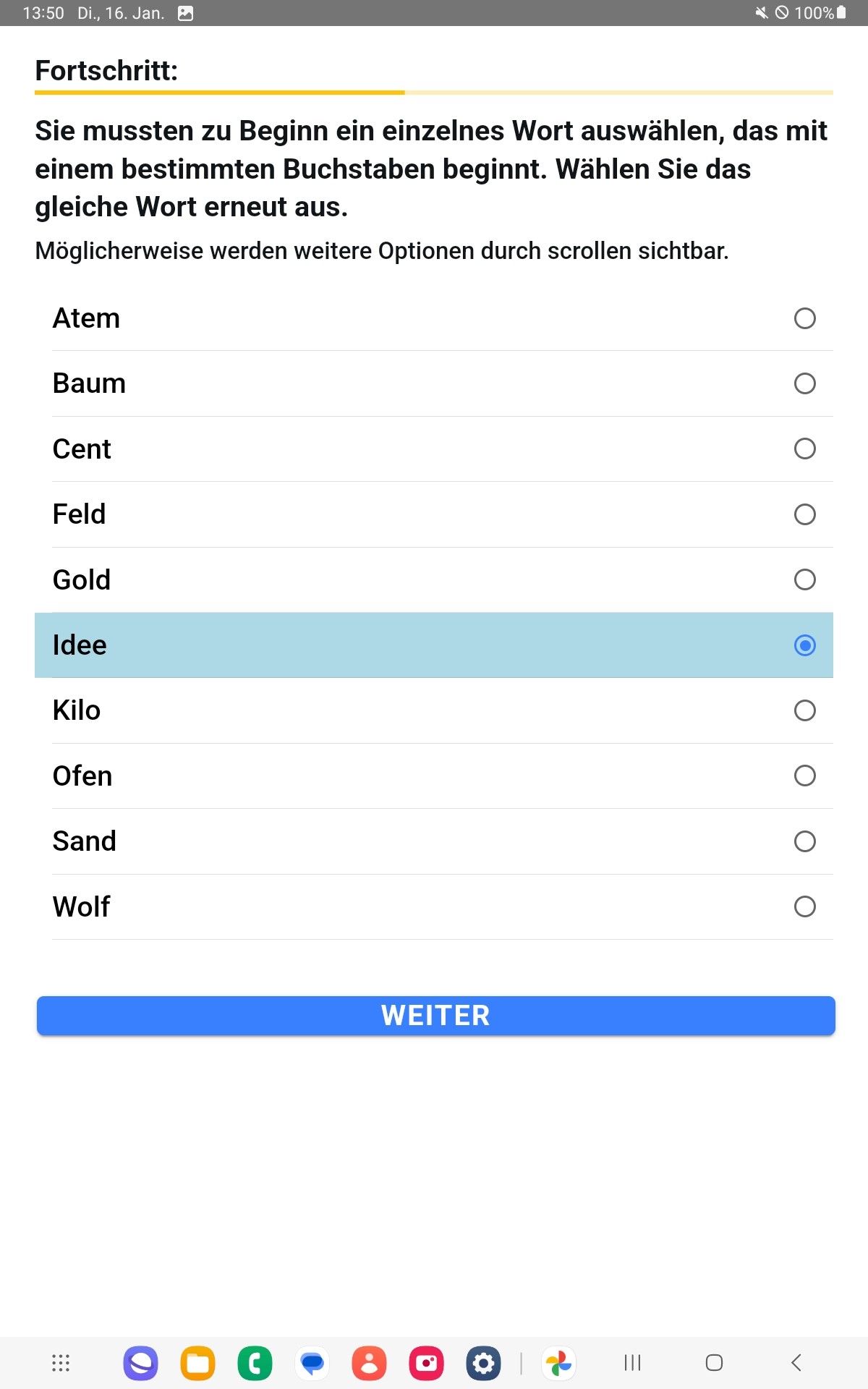


Figure 12: Task 18 (Memory Section)


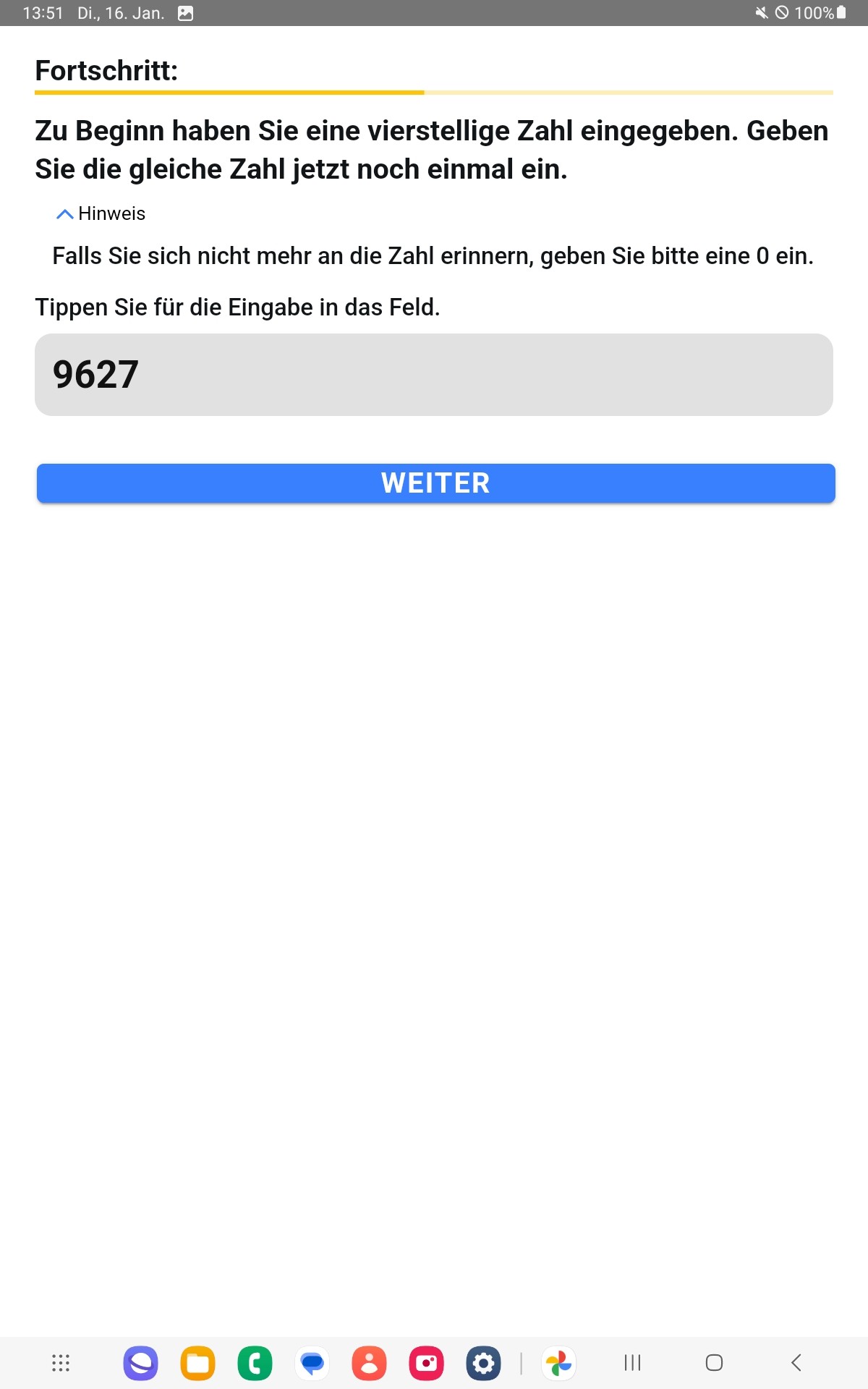


Figure 13: Task 19 (Memory Section)


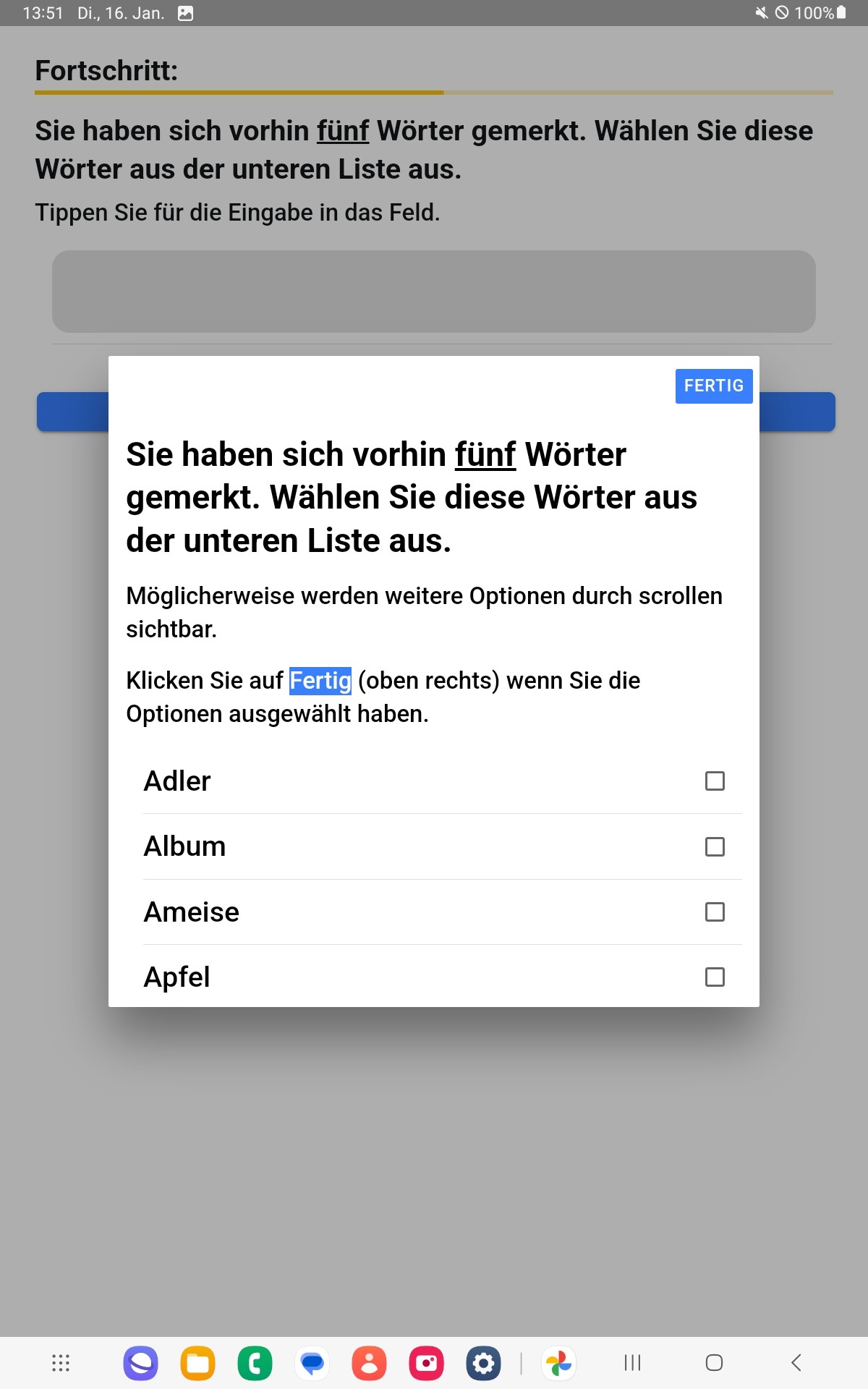


Figure 14: Task 20 (Memory Section)


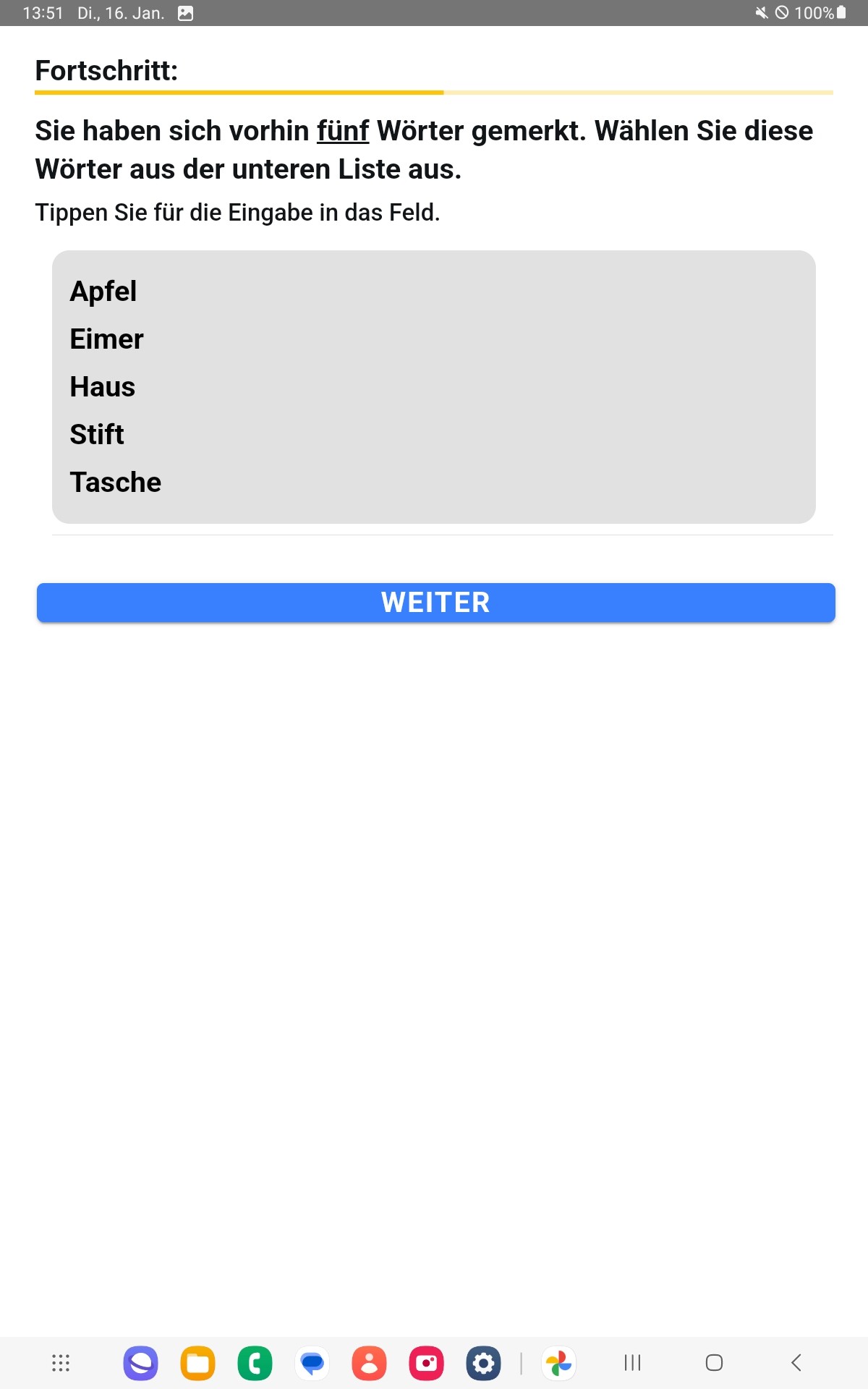


Figure 15: Task 20 / 2 (Memory Section)


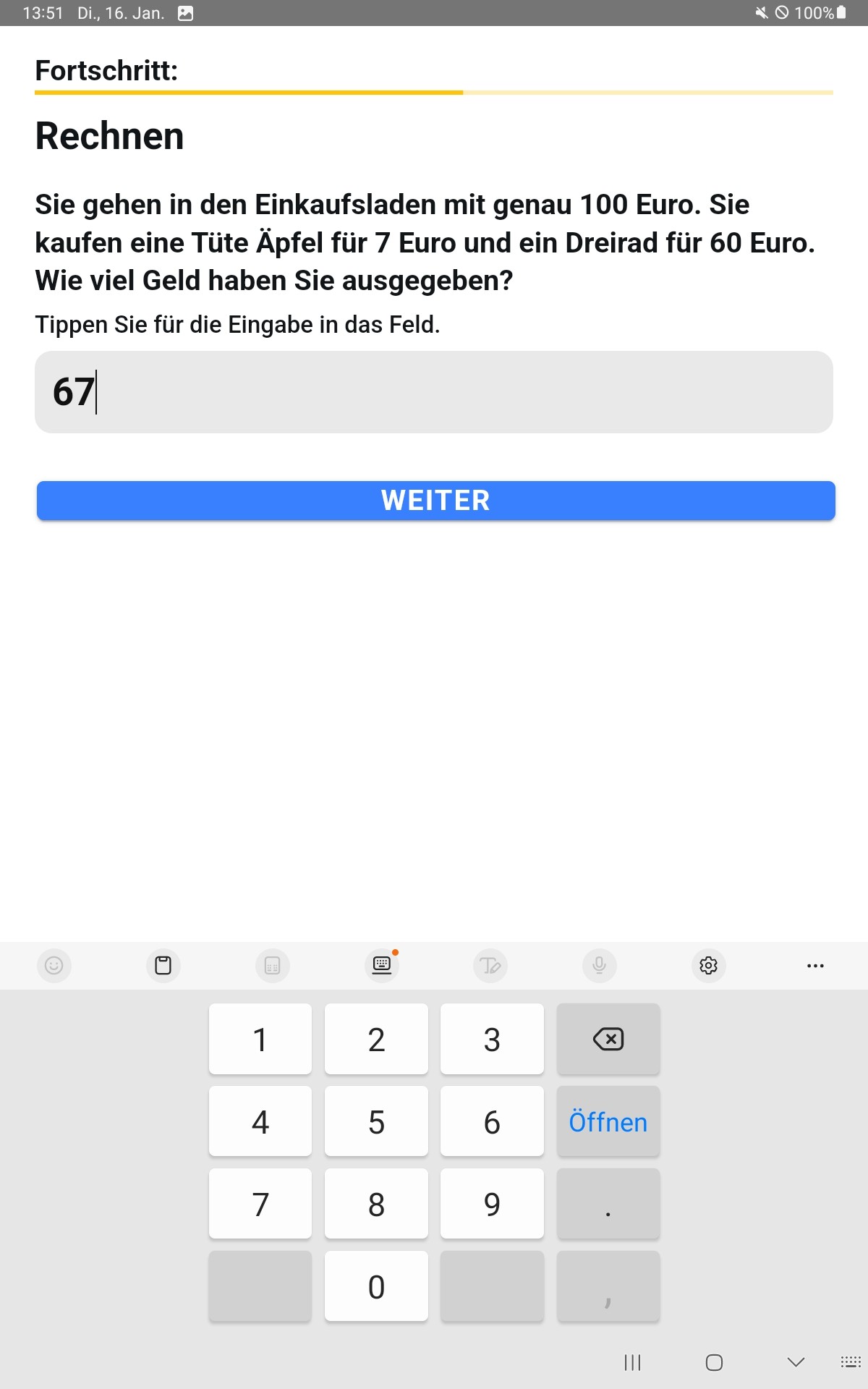


Figure 16: Task 21 (Calculation Section)


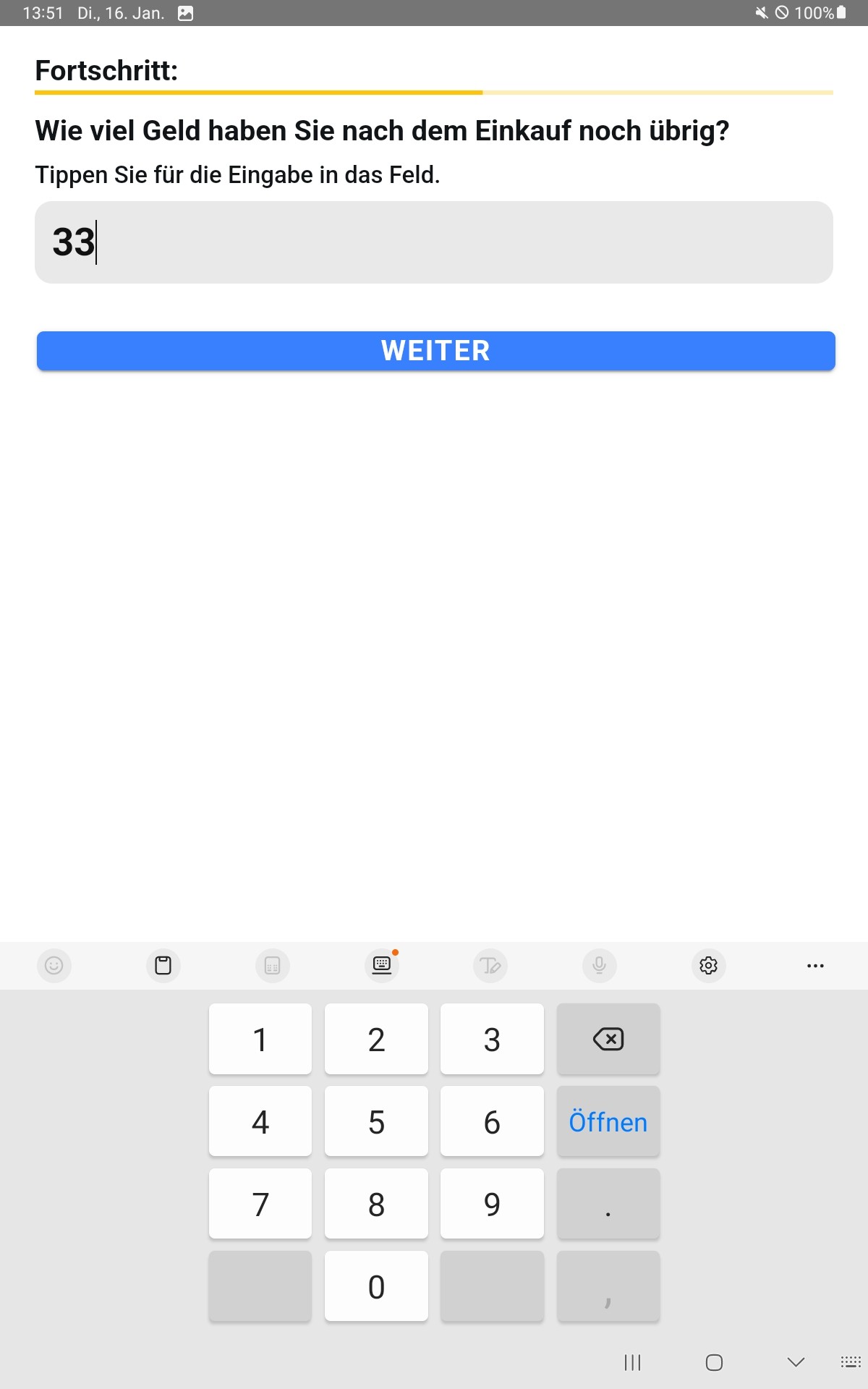


Figure 17: Task 22 (Calculation Section)


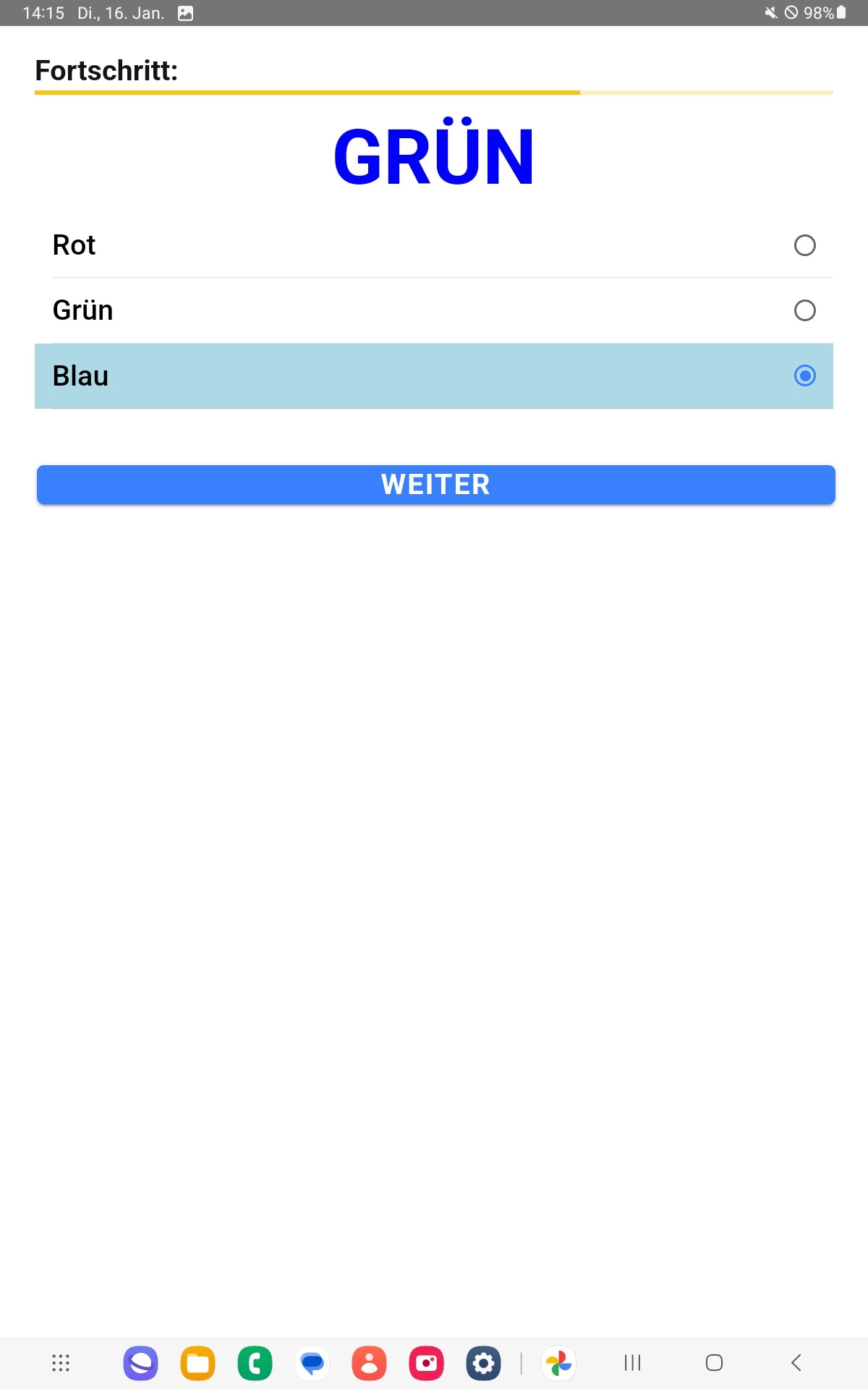


Figure 18: Task 23-34 (Execution Section)


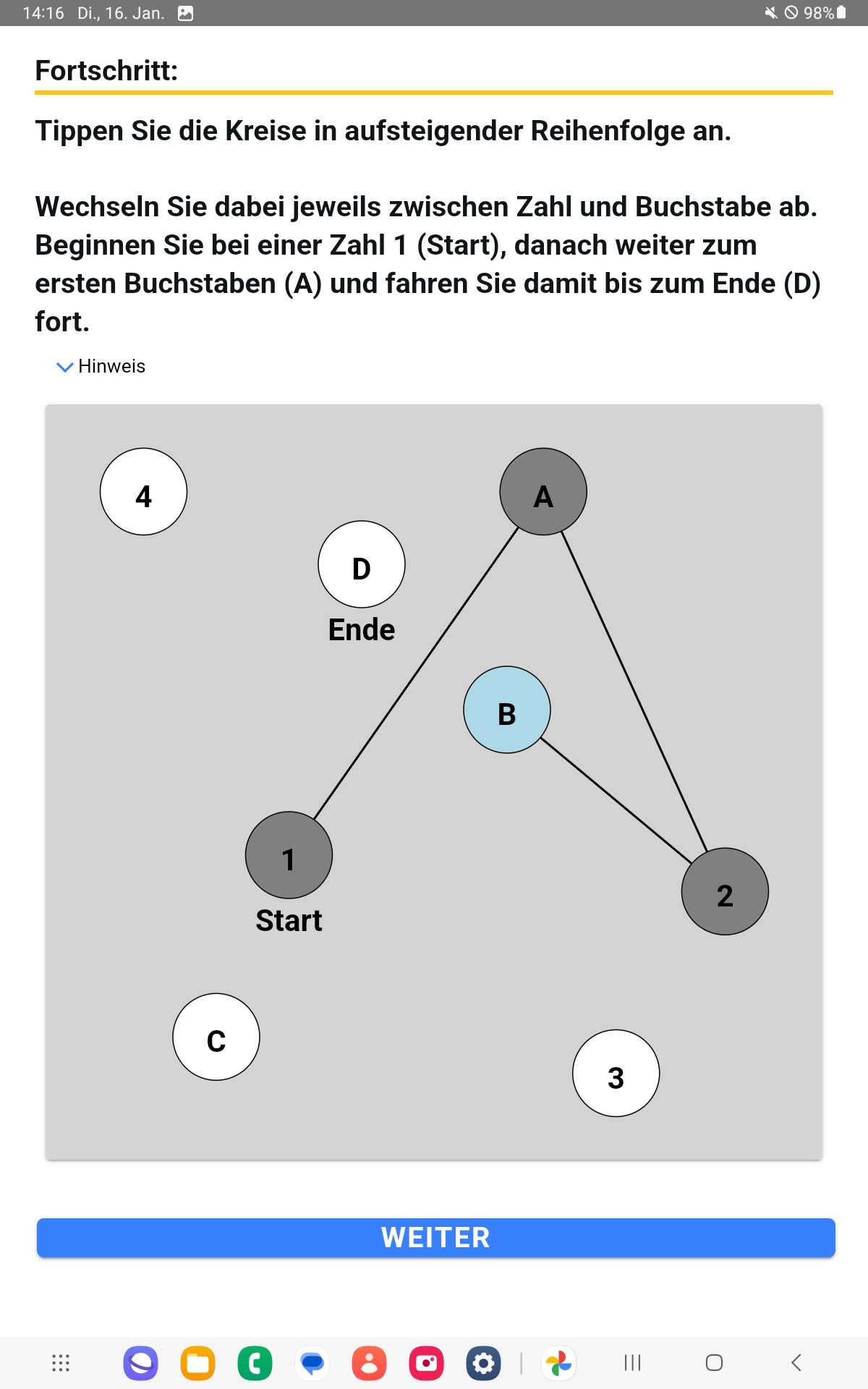


Figure 19: Task 35-36 (Execution Section)
